# Patterns of base chromosome numbers, genome size and rDNA loci evolution in the genus *Capsicum* (Solanaceae)

**DOI:** 10.3389/fpls.2026.1959260

**Published:** 2026-09-28

**Authors:** Vildana Kapetanovic, Carolina Carrizo García, Eva M. Temsch, Rosemarie Benthen, Rebecca Schmidt, Tae-Soo Jang, Hanna Weiss-Schneeweiss

**Affiliations:** 1 Department of Botany and Biodiversity Research, University of Vienna, Vienna, Austria; 2 Instituto Multidisciplinario de Biologia Vegetal, Consejo Nacional de Investigaciones Científicas y Técnicas (CONICET) and Universidad Nacional de Cordoba, Cordoba, Argentina; 3 Department of Biological Science, College of Bioscience and Biotechnology, Chungnam National University, Daejeon, Republic of Korea

**Keywords:** 5S and 35S rDNA loci, ancestral character state reconstruction, base chromosome number, *Capsicum*, evolution, genome size

## Abstract

The size, organization and dynamics of the evolution of plant genomes vary widely among and within evolutionary lineages. The relatively small genus *Capsicum* provides an excellent system for studying genome evolution because all the species are diploid and possess one of two base chromosome numbers (*x* = 12 or 13). Here, we report chromosome numbers, genome sizes, and numbers of 5S and 35S rDNA loci for 24 *Capsicum* taxa representing eight of the nine currently recognized clades and one outgroup species, *Lycianthes rantonnetii*. The size of the analyzed genomes ranged from 1.66 to 7.79 pg/1C (approximately 4.6-fold variation) despite the complete absence of polyploidy. Ancestral state reconstruction based on a RADseq phylogeny inferred a relatively small ancestral genome size (~2.3 pg/1C) for the most recent common ancestor of *Capsicum*, followed by several independent genome expansions and contractions in individual lineages resulting in remarkable genome size uniformity within present-day clades. In the absence of polyploidy, the extent of genome size variation can be attributed to differences in repeatome dynamics. All taxa possessed a single 5S rDNA locus, but the number of 35S rDNA loci varied from one to 18, with signals differing in size and potentially only some loci representing active, intact coding arrays of rDNA monomers. A single major 35S rDNA locus was reconstructed as the ancestral state, followed predominantly by independent increases to two or three major active loci in different lineages. Differences in 35S rDNA loci appearance suggested that the minor signals might represent rDNA-derived tandem repeats, and that 35S rDNA monomers might have independently given rise to novel tandem repeats in different evolutionary lineages of the genus. The ancestral base chromosome number was inferred as *x* = 12, with two independent events of ascending dysploidy (*x* = 12 → 13) during the diversification of two derived, non-sister clades. These findings demonstrate that the evolution of genome size and base chromosome number differs in *Capsicum*, and that major genomic changes coincided with the origin of major clades rather than with the subsequent speciation within the lineages, resulting in strong genomic uniformity within clades.

## 1 Introduction

Plant genomes are very plastic and vary greatly in size and structure. Chromosomal rearrangements, polyploidy, and the dynamics of the repetitive DNA fraction are major processes contributing to genome evolution, diversification, and speciation, with the latter two contributing significantly to genome size variation (He et al., 2024). Polyploidy instantly and proportionally multiplies genome size and chromosome numbers of an organism/taxon, whereas the changes of the repetitive DNA fraction of plant genomes have more gradual effects mostly on the size of the genome, predominantly due to amplification of dispersed elements and increase of copy and loci numbers of tandem repeats. Rampant polyploidy and following periods of cytological and genetic post-polyploidy diploidization accompanied evolution of nearly all angiosperm lineages (Weiss-Schneeweiss et al., 2026). The dynamic interplay of both processes does not only affect the genome size of the organisms and their gene content or expression but is also associated with karyotype restructuring often leading to dysploid chromosome number changes (Weiss-Schneeweiss et al., 2026).

The smallest angiosperm genomes have been reported in a carnivorous Lentibulariaceae, with *Genlisea tuberosa* carrying only 0.065 pg/1C of DNA (Fleischmann et al., 2014), while the largest angiosperm genome of Japanese canopy plant *Paris japonica* (Melanthiaceae) has been estimated to be 2400-fold larger with 152.23 pg/1C of DNA (Pellicer et al., 2010). Genome size dynamics and evolution can vary greatly between and within plant lineages, with both downsizing and upsizing accompanying taxa diversification and speciation (Pellicer et al., 2018; de Tomás and Vicient, 2024). Such variation occurs across temporal and spatial scales, shaped by biotic and abiotic factors, including environmental cues (Bureš et al., 2024; Moreira et al., 2024). Nuclear DNA content is therefore often used as one of the basic diagnostic characters in evolutionary taxonomic studies (McCann et al., 2020; Bureš et al., 2024).

Among the diverse repetitive elements found in plant genomes, rDNAs are unique in being indispensable and integral components of the transcriptome (Goffová and Fajkus, 2021). Both major types of ribosomal DNAs, 35S and 5S rDNAs are housekeeping genes with multiple tandemly arranged copies localized in a number of distinct chromosomal loci. rRNA genes encode rRNA components of the ribosomes and are therefore very conserved in their coding regions and often used as first marker of choice for characterization of the karyotypes of related species via fluorescence *in situ* hybridization (FISH). Conversely, their loci numbers and distribution can vary significantly even among closely related species, and undergo dynamic changes accompanying species diversification on diploid level as well as post-polyploid genome diploidization (Roa and Guerra, 2015; Salim and Gerton, 2019; Hemleben et al., 2021; Rosselló et al., 2022; Wang et al., 2023). Fast evolving spacer regions of both 5S rDNA (non-transcribed spacer, NTS) and 35S rDNA (internal, external and intergenic spacers: ITS1 + 2, ETS, IGS) are widely used for phylogenetic inferences among related taxa (Jang et al., 2018; Rosselló et al., 2022; Wang et al., 2023). These spacers, particularly the IGS region of 35S rDNA monomers, are rich in low complexity repeats and are known to give rise to various tandemly repeated satellite DNAs (Hemleben et al., 2007; Almeida et al., 2012; Hemleben et al., 2021; Rosselló et al., 2022).

An excellent model in which to study evolutionary patterns and dynamics of repetitive DNA fraction and genome size is the genus *Capsicum* in the family Solanaceae. The genus comprises 43 species in nine clades and is native to the Americas with distributions ranging from southern USA to central Argentina and southern Brazil (Carrizo García et al., 2016, 2022; Barboza et al., 2022). Five of the species are domesticated and cultivated as vegetables and spices, with *C*. *annuum* L., *C*. *chinense* Jacq. and *C*. *frutescens* L. cultivated worldwide and *C*. *baccatum* L. and *C*. *pubescens* Ruiz & Pav. cultivated primarily in South America (Barboza et al., 2022; Palombo et al., 2026). Chile peppers are mostly known for their pungency, related to the presence of a unique group of compounds called capsaicinoids (Fattori et al., 2016). All *Capsicum* species are diploid, with two basic chromosome numbers reported, *x* = 12 and *x* = 13 (Scaldaferro et al., 2016; Barboza et al., 2022). Each of the main nine *Capsicum* clades is characterized by the same base chromosome number, with crown group lineages containing domesticated taxa possessing *x* = 12 (Pozzobon and Schifino-Wittmann, 2006; Moscone et al., 2007; Carrizo García et al., 2022). The only two lineages based on *x* = 13 are Atlantic Forest and Andean clades (Barboza et al., 2022). The Andean clade is the most basal lineage of the genus, raising questions about the directionality of dysploidy (ascending vs. descending). Despite shared chromosome number of *x* = 13 and the presence of a much smaller 13^th^ chromosome pair, genomes of the Atlantic Forest and the Andean clades, however, considerably differ in sizes (Barboza et al., 2022). Since chromosomal analyses of *Capsicum* are strongly limited by the near-uniformity of predominantly meta- and submetacentric chromosomes and the lack of sufficient cytogenetic landmarks, the questions of the ancestral chromosome number of the genus and directionality of dysploidy remain unanswered.

Mapping of 35S and 5S rDNA loci in *Capsicum* chromosomes using FISH, combined with analyses of the distribution of GC-rich heterochromatic blocks, allowed for partial karyotype characterization in nearly half of the *Capsicum* species (Scaldaferro et al., 2013, 2016; Scaldaferro and Moscone, 2019; Barboza et al., 2022; de Almeida and Clarindo, 2025) mostly, however, focusing on crown group clades containing cultivated taxa and on the most basal Andean clade (Scaldaferro and Barboza, 2023). One 5S rDNA locus has consistently been mapped in all taxa (Aguilera et al., 2016; Scaldaferro and Moscone, 2019) except for *C*. *regale*, in which three 5S rDNA loci have been identified, two of which were hypothesised to be inactive (Scaldaferro and Barboza, 2023). The number of 35S rDNA loci remained more difficult to elucidate, with the signals varying in strength and ranging from one to 30, further confounded by reported interspecific and intraspecific variation in their number, distribution, and size (Kwon and Kim, 2009; Scaldaferro et al., 2016; Romero-da Cruz et al., 2017; Scaldaferro and Moscone, 2019). The position of many, but not all, 35S rDNA loci in *Capsicum* chromosomes coincided with the GC-rich heterochromatic regions, a pattern observed also in other species due to GC-rich composition of rDNA monomers (Moscone et al., 2007; Scaldaferro et al., 2013; Grabiele et al., 2018). Some of the heterochromatic blocks have, however, also been shown to contain novel satellite DNAs, with no similarity to rDNA monomers (de Assis et al., 2020). The large number of 35S rDNA signals has been suggested to represent not only active loci, but also satellite DNAs amplified from within rDNA monomers. Non-coding regions of 35S rDNA monomers, particularly intergenic spacers (IGS), are known to have given rise to non-coding satellite DNAs independently in several unrelated plant lineages, including members of the Solanaceae family (Jo et al., 2009, 2011; Sader et al., 2021). Such amplification of new satellite DNA may not only affect overall genome size of the species but also makes unambiguous identification of active rDNA loci difficult (Grabiele et al., 2018; Scaldaferro and Moscone, 2019). The evolution of satellite DNA(s) from within the 35S rDNA monomer has been suggested to have occurred in cultivated species of *Capsicum*, however, the independent evidence suggested very different scenarios (Jo et al., 2011; Grabiele et al., 2018), with one study inferring presence of “megasatellite DNA” derived from rDNA, further complicating the interpretation of the observed patterns (Grabiele et al., 2018).

Genome sizes have so far been reported for 17 *Capsicum* taxa (14 species and three varieties) with the known 1C genome size values varying between 2.08 pg – 7.2 pg, suggesting c. 3.4-fold difference (Belletti et al., 1998; Moscone et al., 2003; Barboza et al., 2022). The reported values, however, are partly inconsistent, especially, but not only, in cultivated species and incomplete with taxonomic sampling strongly biased towards crown group clades. This may reflect natural variation within species, or the impact of human-assisted selection, but can also stem from the use of different methods to obtain the data. Thus, a systematic evaluation of genome sizes and their evolution is needed to understand the patterns of genome size variation and its role in *Capsicum* diversification, speciation and evolution (Belletti et al., 1998; Moscone et al., 2003; Jarret et al., 2019).

Given the apparent absence of polyploidy in *Capsicum*, the genome size evolution in this genus is hypothesized to be primarily driven by dynamic changes in repetitive DNA fraction, including but not limited to rDNAs. It is also hypothesized that patterns and dynamics of the base chromosome number and genome size evolution differ. Thus, the aims of the current study are: (1) to report chromosome numbers, karyotypes, and genome sizes, and 5S and 35S rDNA loci number and distribution in mitotic metaphase chromosomes of 22 species and three varieties of *Capsicum* and one species of sister genus *Lycianthes*, using data collected from the same individuals to allow for unambiguous and direct comparison across character types; (2) to reconstruct the evolution of genome size, base chromosome number, and 35S rDNA loci number using a RADseq-based genus-wide phylogeny (Carrizo García et al., 2022); and (3) to use this framework to address competing hypotheses regarding the directionality (ascending vs. descending) and origin (single vs. recurrent) of the two base chromosome numbers found in the genus. For several of the species some or all of these data are reported here for the first time, complementing existing records and providing an integrated phylogenetic framework to explore genome size and rDNA loci number evolution across the genus.

## 2 Materials and methods

### 2.1 Plant material

Twenty-two *Capsicum* species and three varieties of *C. baccatum* representing eight major clades of the genus and one outgroup species, *Lycianthes rantonnetii* were analyzed (**Table 1**). All plant material was collected from natural populations or obtained from commercial sources (**Supplementary Table 1**) and subsequently cultivated outdoors or in the greenhouse (in winter) in the Botanical Garden of the University of Vienna, Austria.

**Table 1:** Chromosome numbers, numbers and types of 35S and 5S rDNA loci and genome sizes of analyzed species of *Capsicum* and of *Lycianthes rantonnetii*.

Taxon	Population/individual	2*n*	Number of 35S and 5S rDNA loci*		Genome size 1C (pg) +SD	Figures
			35S	5S		
Annuum clade						
*C. annuum* L. var. *annuum* (1)	50	24	2 [major]	1	3.49 (± 0.007)	1, 2A, 4
*C*. *annuum* L. var. *annuum* (2)	20141	24	1 [major]	1	3.46 (± 0.015)	1, 2B, 4
*C*. *chinense* Jacq.	2040	24	11 [6 major; 5 minor]	1	3.78 (± 0.045)	1, 2C, 4
*C. frutescens* L.	2064	24	8 [2 major; 6 minor]	1	3.43 (± 0.015)	1, 2D, 4
*C*. *galapagoense* Hunz.	59	24	11 [4 major; 7 minor]	1	3.45 (± 0.004)	1, 2E, 4
Baccatum clade						
*C*. *baccatum* L. var. *baccatum*	GEB 1807	24	15 [3 major; 12 minor]	1	3.73 (± 0.021)	1, 2F, 4
*C*. *baccatum* var. *pendulum* (Willd.) Eshbaugh	3642	24	15 [5 major; 10 minor]	1	3.74 (± 0.013)	1, 2G, 4
*C*. *baccatum* var. *umbilicatum* (Vell.) Hunz. & Barboza	390	24	10 [4 major; 6 minor]	1	3.79 (± 0.038)	1, 2H, 4
*C*. *chacoense* Hunz.	90	24	2 [major]	1	3.52 (± 0.016)	1, 2I, 4
*C*. *rabenii* Sendtn.	CGN 22795	24	13 [5 major; 8 minor]	1	4.24 (± 0.028)	1, 2J, 4
Pubescens clade						
*C*. *cardenasii* Heiser & P.G.Sm.	3656	24	17 [9 major; 8 minor]	1	4.47 (± 0.035)	1, 2K, 4
*C*. *eshbaughii* Barboza	67	24	8 [2 major; 6 minor]	1	4.06 (± 0.041)	1, 2L, 4
*C. eximium* Hunz.	14	24	13 [5 major; 8 minor]	1	4.28 (± 0.090)	1, 2M, 4
*C*. *pubescens* Ruiz & Pav.	56	24	12 [2 major; 10 minor]	1	4.48 (± 0.019)	1, 2N, 4
Tovarii clade						
*C*. *tovarii* Eshbaugh, P.G.Sm. & Nickerent	57	24	14 [3 major; 11 minor]	23	5.36 (± 0.144)	1, 3A, 4
Bolivian clade						
*C. caballeroi* M.Nee	420	24	1 [major]	1	6.15 (± 0.011)	1, 3B, 4
*C. minutiflorum* (Rusby) Hunz.	460	24	1 [major]	1	5.97 (± 0.015)	1, 3C, 4
Flexuosum clade						
*C*. *flexuosum* Sendtn.	430	24	18 [2 major; 16 minor]	1	7.79 (± 0.202)	1, 3D, 4
Atlantic Forest clade						
*C. recurvatum* Witasek	1632	26	2 [major]	1	4.28 (± 0.008)	1, 3E, 4
*C. schottianum* Sendtn.	380	26	2 [major]	1	4.20 (± 0.007)	1, 3F, 4
Andean clade						
*C. geminifolium* Barboza & Leiva	AOR2688	26	1 [major]	1	1.87 (± 0.001)	1, 3G, 4
*C. lanceolatum* (Greenm.) C.V.Morton & Standl.	51	26	1 [major]	1	1.66 (± 0.001)	1, 3H, 4
*C. longifolium* Barboza & Leiva	4851	26	1 [major]	1	2.02 (± 0.009)	1, 3I, 4
*C*. *lycianthoides* (Dammer) Hunz.	Beltrán 85	26	1 [major]	1	1.96 (± 0.030)	1, 3J, 4
*C. rhomboideum* (Dunal) Kuntze	Garcia 20	26	1 [major]	1	1.96 (± 0.004)	1, 3K, 4
Outgroup						
*L. rantonnetii* (Carrière) Bitter	s.n.	24	1 [major]	1	1.30 (± 0.008)	3L, 4

*Minor loci refer to loci that are small, but also those that are larger but diffuse in appearance and do not form clearly defined blocks in chromosomes.

### 2.2 Chromosome preparation

Actively growing root-tips of all analyzed taxa, each taxon represented by one individual, were harvested and pretreated with 0.002 M 8-hydroxyquinoline (Sigma, Aldrich, Vienna, Austria) for 2.5 hours at room temperature followed by 2.5 hours at 4 °C. The samples were fixed in a fixative (3: 1 v/v; ethanol: acetic acid) and stored at -20 °C until use. Chromosome numbers and karyotypes were determined using standard Feulgen staining (Jang et al., 2013). Briefly, root meristems were incubated in 5N HCl (Sigma Aldrich, Vienna, Austria) at room temperature for 12 minutes, washed with tap water and stained with Schiff’s reagent (Merck, Darmstadt, Germany) for 60 minutes in darkness at room temperature. Meristems were dissected on a slide in a drop of 60% acetic acid and squashed under the coverslip. Slides were analyzed using the light microscope Axio Imager M2 (Carl Zeiss, Vienna, Austria) equipped with a digital camera. Well-dispersed and complete metaphase plates were photographed using ZEN software (Carl Zeiss, Vienna, Austria). Minimum of ten metaphase cells were analyzed per species. Chromosomes were cut-out from the images and karyotypes were constructed using Corel Photo Paint (Corel Corporation, Ottawa, Canada). Due to high levels of similarities between individual chromosome size and type that hampered unambiguous identification of individual pairs, the localization of 5S and 35S rDNA loci in haploid chromosome sets was summarized as ideograms with all chromosomes represented by uniformly sized bars (generalized ideograms), without indicating the position of the centromeres. The only exception was the 13^th^ chromosome of *x* = 13 lineages that was significantly smaller.

Chromosomal spreads for FISH were prepared by enzymatic digestion and squashing with minor modifications (Weiss-Schneeweiss and Jang, 2023). Briefly, meristems were digested for 18 minutes at 37 °C with enzyme cocktail (1% cellulase Onozuka, Serva, Heidelberg, Germany; 0.4% cytohelicase, Sigma-Aldrich, Vienna, Austria; and 0.4% pectolyase, Sigma-Aldrich, Vienna, Austria) in citric buffer. Digested material was transferred onto a slide, dissected in a drop of 60% acetic acid and gently squashed. The quality of the preparations was checked under a phase-contrast microscope Axio Imager M2 (Carl Zeiss, Vienna, Austria). Coverslips were removed at -80 °C and the preparations were air-dried. Probes used for FISH were 35S rDNA (coding regions of 18S and/or 25S) from *Arabidopsis thaliana* in plasmid pSK+, 5S rDNA (coding region) from *Melampodium montanum* in plasmid pGEM‐T Easy, as well as commercially synthesized (Sigma Aldrich, Vienna, Austria) pooled oligoprobes representing conserved regions of 18S, 5.8S and 25S rDNAs (Waminal et al., 2018). Probes were labeled with biotin or digoxigenin (Roche, Vienna, Austria) either directly by PCR (5S rDNA), using a nick translation kit (35S rDNA; Roche, Vienna, Austria) or were commercially labelled (oligoprobes). Antidigoxigenin antibody conjugated with FITC (Sigma-Aldrich, Vienna, Austria) was used to detect digoxigenin and ExtrAvidin conjugated with Cy3 (Sigma-Aldrich, Vienna, Austria) was used to detect biotin. Re-fixation, RNase and pepsin treatments, denaturation, stringent washes and antibody detection were performed as described previously (Weiss-Schneeweiss and Jang, 2023). Preparations were analyzed with an Axiolmager M2 epifluorescence microscope (Carl Zeiss, Vienna, Austria). Images of multiple well-spread and complete metaphase plated were captured with a CCD camera and processed using ZEN software (Carl Zeiss, Vienna, Austria) with only those functions that apply to all pixels of the image equally. Minimum of ten metaphase spreads were analyzed per species.

### 2.3 Genome size estimation using flow cytometry

Genome sizes of 24 analyzed *Capsicum* taxa and of *Lycianthes rantonnetii* were measured using flow cytometry with *Solanum pseudocapsicum* L. (1C = 1.29 pg, Temsch et al., 2010) as the internal standard. Twenty-five mg of freshly collected leaf tissue was co-chopped with leaves of the standard in Otto’s buffer I (Otto et al., 1981; Galbraith et al., 1983). The cell suspension was filtered through a 30 µm nylon mesh, incubated with DNase-free RNaseA at 37 °C (final concentration of 0.15 mg/mL; Sigma Aldrich, Vienna, Austria) for 30 minutes and stained in Otto’s buffer II (Otto et al., 1981) containing 60 mg/L propidium iodide (PI; AppliChem GmbH, Germany) for 1 hour or overnight at 4 °C. Genome sizes were measured using CyFlow ML cytometer (Sysmex Partec GmbH, Germany) equipped with diode-pumped laser (532 nm, 100 mW, Cobolt AB, Sweden). Ten thousand particles were measured per preparation (exceptionally 3,333 in rare cases) and each sample was measured in three technical replicates. The CVs of all individual runs/replicates were below 3%, with most ranging from 1.2% to 2.5%. The 1C values of samples were calculated using the standard equation: 1C-value _sample_ = mean FI _sample G1_/mean FI _standard G1_ × 1C-value _standard_.

### 2.4 Ancestral state reconstructions and character evolution

Ancestral states were reconstructed for three characters: genome size (1C), base chromosome number (*x*), and the number of major 35S rDNA loci. 5S rDNA loci numbers were not used for reconstruction given a conserved and consistent number of loci in all species analyzed. Only data generated in this study were used for evolutionary reconstructions of genome sizes and 35S rDNA loci since all the data points were obtained from the same individuals, thus eliminating the error that could stem from natural variation within taxa. Base chromosome number reconstruction was based on both current and previously published data, to maximize the sample size (**Table 1**; **Supplementary Table 2**). All analyses and tree annotations were performed in RStudio (R Core Team, 2026). Input trees for ancestral state reconstructions were taken from Carrizo García et al. (2022) and adapted to the current datasets by excluding taxa with missing data. To this end, tips with missing data were removed from the input tree using the function drop.tip of the ape package (Paradis and Schliep, 2019).

Genome size values (1C) measured in picograms (pg) were treated as a continuous trait. The fitContinuous function of the geiger package (Harmon et al., 2008) was used to compare the best-fitting evolutionary model among Brownian Motion (BM), early burst (EB), and Ornstein-Uhlenbeck (OU), following the Akaike Information Criterion (AIC). AIC values were ranked according to their weights, calculated using the function aic.w in geiger (Pennell et al., 2014). Although BM and EB had very similar AIC weights, ancestral states were inferred setting the best-fitting BM model (**Supplementary Data 1**) using the function anc.ML in phytools (Revell, 2012). Ancestral states were additionally reconstructed under EB to evaluate robustness. Both models yielded qualitatively identical reconstructions; therefore, only the BM-based reconstructions are presented in the Results.

Both base chromosome number and 35S rDNA loci numbers were coded as discrete traits. The base chromosome number represented two states, *x* = 12 and *x* = 13. Given the extent of 35S rDNA signal number variation in the analyzed species and the difficulty in unambiguous determination of active coding 35S rDNA loci numbers, the data matrix was simplified to three states to avoid overinterpretation of the data: species with one locus, species with two loci, and species with three or more loci (correspondingly states of 1, 2 or ≥ 3). 35S rDNA loci were classified as major or minor based on their relative signal intensity and size across cells within each species, using the maximum number of active 35S rDNA loci reported in the literature as an additional guideline. The best fitting model for the two discrete characters was evaluated using the fitDiscrete function of the geiger package, contrasting different models for different datasets. For the binary character base chromosome number, four transition models were tested to evaluate the competing hypotheses of ascending versus descending dysploidy (**Supplementary Data 1**, **2**): single Equal Rate (ER), All (backward and forward) Rates Different (ARD), and two irreversible models restricting transitions either from 12 to 13 (Irr-1, consistent with ascending dysploidy) or from 13 to 12 (Irr-2, consistent with descending dysploidy).

For the three-state 35S rDNA loci numbers, four models were contrasted to determine whether locus number evolution follows an ordered or unordered process (**Supplementary Data 1**, **2**). The unordered models allowed direct transitions between non-consecutive states under equal rates (ER), fully free rates (ARD), or symmetrical rates (SYM) between pairs (equal forward and reverse but potentially different between pairs). In contrast, the ordered meristic model (MER) assumed transitions between consecutive states only (i.e., preventing intermediate steps from being skipped) with unequal transition rates between steps. The best fitting model was selected for both discrete characters following the AIC, as described above. The best fitting models were Irr-1 for the base chromosome number, and ER for the number of 35S rDNA loci numbers (**Supplementary Data 1**, **2**). Ancestral states for these two characters were reconstructed via stochastic character mapping (Huelsenbeck et al., 2003) using the function make.simmap of phytools. The transition model was specified for each character according to the best fitting model selected. In all cases, 500 simulations were run with the argument pi set as estimated; the stochastic maps obtained were summarized using the phytools function describe.simmap. Trees were annotated with the results of the different reconstructions using the ggtree package (Yu et al., 2017).

An additional analysis to reconstruct base chromosome number evolution was performed using ChromEvol v2.0 (Glick and Mayrose, 2014). ChromEvol was ran with default homogeneous models, allowing the software to automatically select the best-fitting model based on AIC. The six default models were evaluated: Dys (dysploidy only; ascending + descending dysploidy, constant rates), DysDup (dysploidy + whole-genome duplication, constant rates), DysDupDem (dysploidy + duplication + demi-duplication, all rates constant and estimated independently), DysDupDem= (dysploidy + duplication + demi-duplication, with demi-duplication rate constrained equal to duplication rate), DysBnum (dysploidy + base-number addition, addition of multiples of x, constant rates), and DysBnumDup (dysploidy + base-number addition + duplication, constant rates). The Dys model (dysploidy only, no polyploidy or base-number addition) was selected as best-fitting (AIC 22.07).

The phylogenetic signal (i.e., inference whether more closely related species tend to display similar character states) was quantified for all three characters. The Pagel’s λ (Pagel, 1999) and Blomberg’s *K* (Blomberg et al., 2003) were estimated to evaluate the phylogenetic signal for the genome size, using the phylosig function of the phytools package for the genome size. Pagel’s λ varies continuously between 0 and 1, with 0 indicating no phylogenetic signal and 1 strong phylogenetic signal. A value of Blomberg’s *K* = 1 indicates that the trait has evolved under a Brownian motion model; *K* < 1 suggests less phylogenetic signal than expected, while *K* > 1 indicates a strong phylogenetic signal, meaning close relatives are more similar in genome size than expected under Brownian motion. The statistical significance of both metrics was assessed using a randomization test with 1, 000 simulations. The phylogenetic signal for the discrete traits was evaluated using two complementary approaches. For the binary base chromosome number, the D statistic (Fritz and Purvis, 2010) was calculated using phylo.d in caper (Orme et al., 2012), with 1, 000 permutations, where D = 1 represents a random distribution across the tree, D = 0 Brownian motion, and D < 0 indicates highly clumped or conserved traits. Additionally, both discrete traits were analyzed with Phylogenetic Generalized Linear Mixed Models (PGLMM) (Hadfield and Nakagawa, 2010) using the package MCMCglmm (Hadfield, 2010). The MCMC chains (n = 4) ran for 110, 000 iterations (thinning = 100, burn-in = 10, 000) on an ultrametric tree (matching tips already adjusted) generated via penalized likelihood via chronos in ape. The phylogenetic signal was quantified as phylogenetic heritability (H^2^) on the latent scale, reporting the posterior mode and 95% highest posterior density (HPD) intervals. Convergence was assessed via effective sample size (ESS > 200) for H².

## 3 Results

### 3.1 Chromosome number and genome size analyses

Chromosome numbers were determined for 24 wild and cultivated taxa of the genus *Capsicum*, representing eight of the nine currently recognized clades (**Table 1**) and one species from the sister genus *Lycianthes*. Diploid chromosome numbers of 2*n* = 24 (*x* = 12) and 2*n* = 26 (*x* = 13) were observed (**Figure 1**; **Supplementary Figure 1**). Species belonging to the Annuum, Baccatum, Tovarii, Bolivian, and Flexuosum clades all possessed chromosome numbers of 2*n =* 24. In contrast, species of the Atlantic Forest and Andean clades all had chromosome numbers of 2*n* = 26. No evidence of polyploidy was detected in any of the species examined. *Lycianthes rantonnetii* had a chromosome number of 2*n* = 24. The chromosome number of 2*n* = 24 (*x* = 12) is reported here for the first time for *C. minutiflorum*.

**Figure 1:**
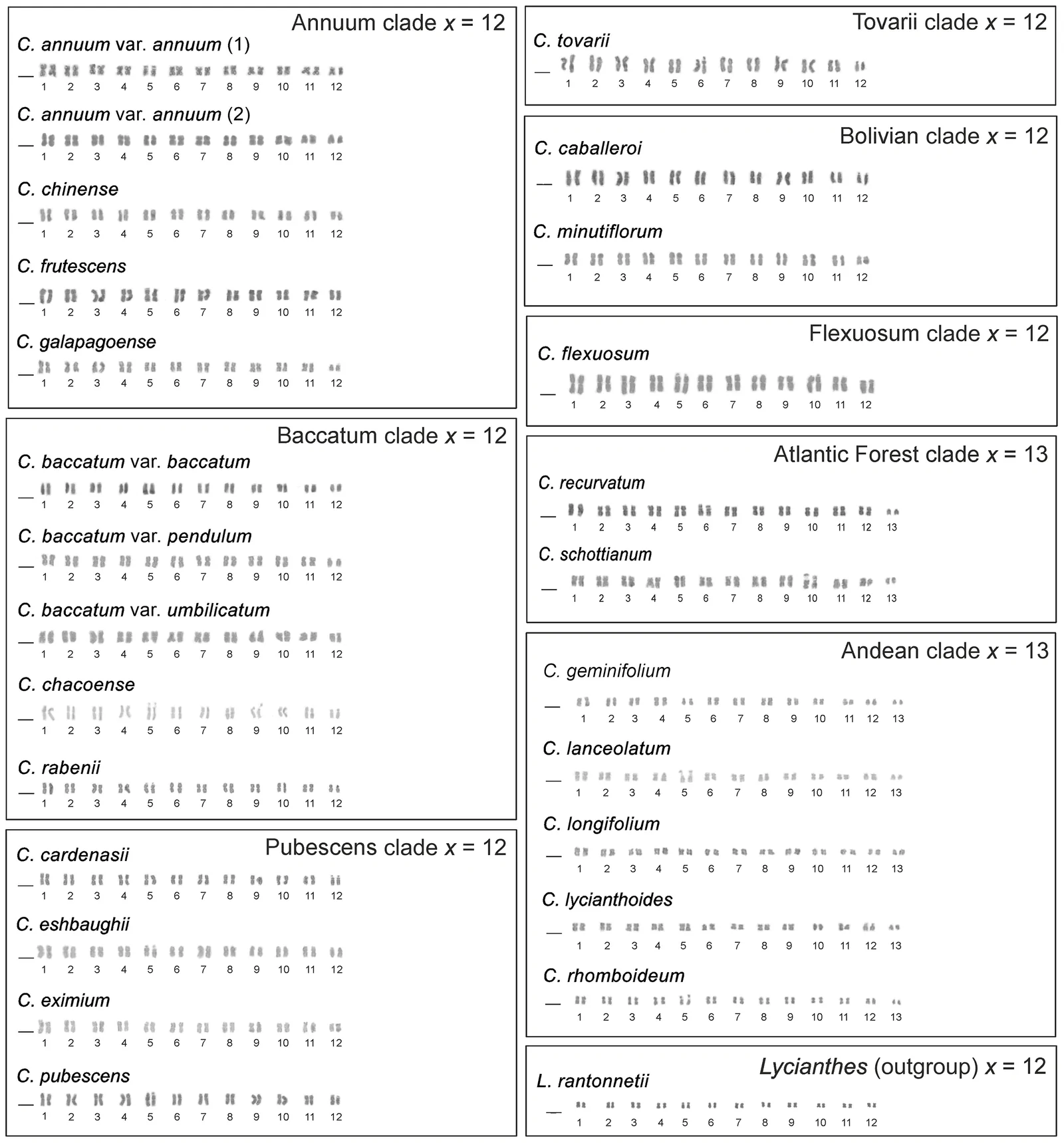
Karyotypes of analyzed *Capsicum* species and the outgroup *Lycianthes rantonnetii* based on mitotic metaphase chromosome spreads. Scale bar, 5 μm.

Flow cytometry was used to determine the nuclear genome sizes of all analyzed samples with *Solanum pseudocapsicum* as an internal reference standard (**Table 1**). This study provides the first genome size measurements for 11 species: *C. caballeroi*, *C. eshbaughii*, *C. galapagoense*, *C. geminifolium*, *C. lanceolatum*, *C. longifolium*, *C. lycianthoides*, *C. minutiflorum*, *C. recurvatum*, *C. schottianum* and *Lycianthes rantonnetii*. The 1C-values ranged from 1.66 pg in *C. lanceolatum* (Andean clade) to 7.79 pg in *C. flexuosum* (Flexuosum clade), representing a 4.6-fold range of genome size within the genus. Species within each clade possessed very similar genome sizes. Species of the Annuum clade, including three cultivated taxa (*C. annuum*, *C. chinense*, *C. frutescens*), had genome sizes ranging from 3.43 pg to 3.78 pg (1C). Three varieties of cultivated *C. baccatum* (var. *baccatum*, var. *pendulum*, var. *umbilicatum*) of the Baccatum clade had genome sizes between 3.73 pg and 3.79 pg, whereas nuclear DNA amounts of their wild relatives, *C. chacoense* and *C. rabenii* were 3.52 pg and 4.24 pg, respectively. Four species of the Pubescens clade had genome sizes ranging from 4.06 pg in *C. eshbaughii* to 4.47 pg in *C. cardenasii*. Genome sizes of *C. tovarii* and *C. flexuosum*, each representing a monotypic clade, were significantly larger with 5.36 pg and 7.79 pg, respectively. Larger genome sizes were also reported here for the first time for *C. caballeroi* and *C. minutiflorum* of the Bolivian clade, with 6.15 pg and 5.97 pg, respectively. Very similar genome sizes (4.28 pg and 4.20 pg) were measured in two representatives of the Atlantic Forest clade, *C. recurvatum* and *C. schottianum*, both reported here for the first time. Genome sizes of five species of the most basal lineage of the genus, the Andean clade, were measured, four of them for the first time. Their genome sizes were very small ranging from 1.66 pg in *C. lanceolatum* to 2.02 pg in *C. longifolium*. The genome size of 1.30 pg of the analyzed outgroup species, *L. rantonnetii* was the smallest measured in this study.

### 3.2 Mapping of 5S and 35S rRNA genes

Both 35S and 5S rDNAs were mapped in mitotic metaphase chromosomes of all 24 analyzed taxa to estimate their loci number and localization (**Table 1**; **Figures 2**–**4**). rDNA loci numbers have been reported here for the first time for *C*. *caballeroi*, *C*. *eshbaughii*, *C*. *galapagoense*, *C*. *minutiflorum*, and *C*. *schottianum*, as well as for the outgroup species *L. rantonnetii* (**Figures 2**–**4**). The number and intensity of 35S rDNA hybridization signals varied significantly within individual genomes, indicating presence of one, two, or three or more loci (**Table 1**). In species with three or more loci, the differences in loci sizes ranged from large through medium to small and very small, and these were therefore assigned to two categories: major and minor. Since the numbers of minor loci, some on the border of detection limit, varied slightly within single individuals, the most representative chromosomal spreads and numbers of minor loci were reported (**Figures 2**–**4**; **Table 1**). The number of 5S rDNA loci was constant throughout the genus, with one locus in the distal region of one chromosome pair. The only exception was *C*. *tovarii*, where larger signals were localized in distal regions of all chromosomes (**Figure 3A**). These signals also appeared less compact than 5S rDNA signals in the remaining species, and it was therefore difficult to unambiguously conclude how many and which of these represented active 5S rDNA locus/loci in *C. tovarii*.

**Figure 2:**
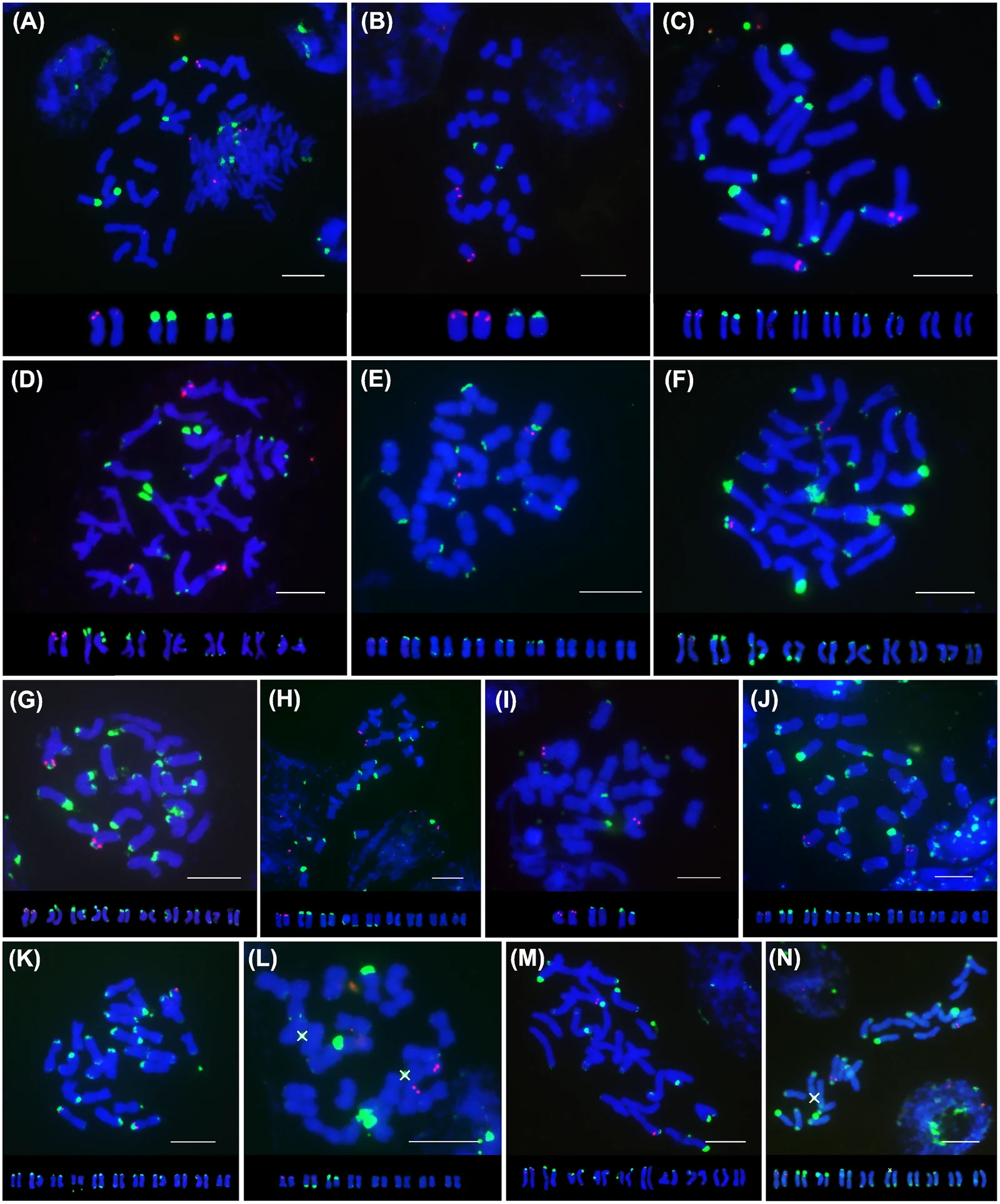
Localization of 35S and 5S rDNAs in mitotic metaphase chromosomes of the *Capsicum* species representing Annuum, Baccatum and Pubescens clades. **(A–E)** Annuum clade, 2*n* = 24: **(A)** *C*. *annuum* var. *annuum* (1), **(B)** *C*. *annuum* var. *annuum* (2), **(C)** *C*. *chinense*, **(D)** *C*. *frutescens*, **(E)** *C*. *galapagoense*. **(F–J)** Baccatum clade, 2*n* = 24: **(F)** *C*. *baccatum* var. *baccatum*, **(G)** *C*. *baccatum* var. *pendulum*, **(H)** *C*. *baccatum* var. *umbilicatum*, **(I)** *C*. *chacoense*, **(J)** *C*. *rabenii*. **(K–N)** Pubescens clade, 2*n* = 24. **(K)** *C*. *cardenasii*, **(L)** *C*. *eshbaughii*, **(M)** *C*. *eximium*, **(N)** *C*. *pubescens*. 5S rDNA signals in red, 35S rDNA in green. Chromosomes carrying signals are cut out and arranged in descending signal strength. “×” in L and N indicates background signals (satellite segments distal to the 35S rDNA loci detached from other chromosomal spreads/cells). Scale bar, 5 μm.

**Figure 3:**
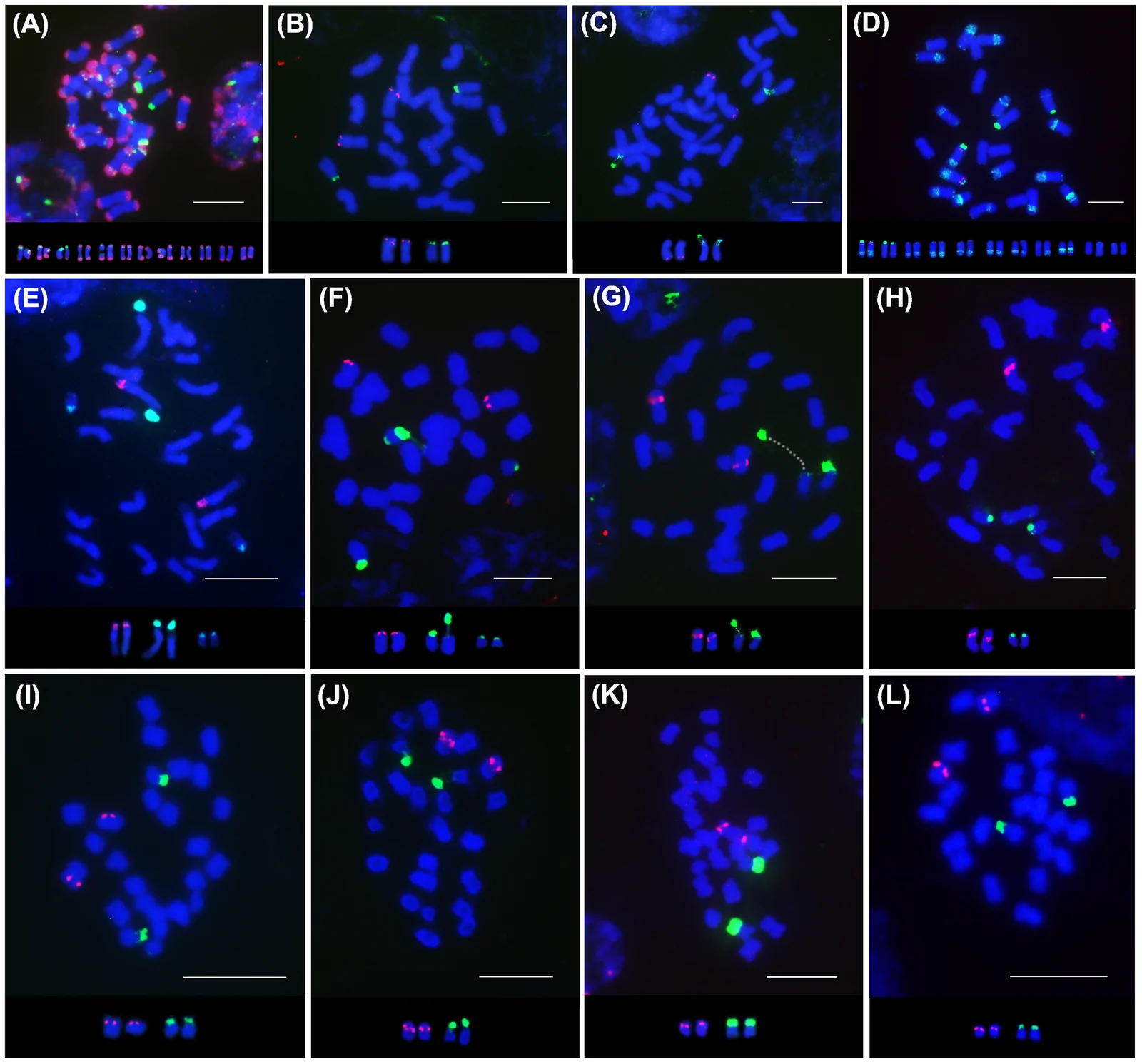
Localization of 35S and 5S rDNAs in mitotic metaphase chromosomes of the *Capsicum* species representing Tovarii, Bolivian, Flexuosum, Atlantic Forest and Andean clades, and of the outgroup species *Lycianthes rantonnetii*. **(A)** Tovarii clade, 2*n* = 24: **(A)** *C*. *tovarii*. **(B, C)** Bolivian clade, 2*n* = 24: **(B)** *C*. *caballeroi*, **(C)** *C*. *minutiflorum*. **(D)** Flexuosum clade, 2*n* = 24: **(D)** *C*. *flexuosum*. **(E, F)** Atlantic Forest clade, 2*n* = 26: **(E)** *C*. *recurvatum*, **(F)** *C*. *schottianum*. **(G–K)** Andean clade, 2*n* = 26: **(G)** *C*. *geminifolium*, **(H)** *C*. *lanceolatum*, **(I)** *C*. *longifolium*, **(J)** *C*. *lycianthoides*, **(K)** *C*. *rhomboideum*. **(L)** Outgroup, 2*n* = 24: **(L)** *Lycianthes rantonnetii*. 5S rDNA signals in red, 35S rDNA in green. Chromosomes carrying signals are cut out and arranged in descending signal strength. Scale bar, 5 μm.

**Figure 4:**
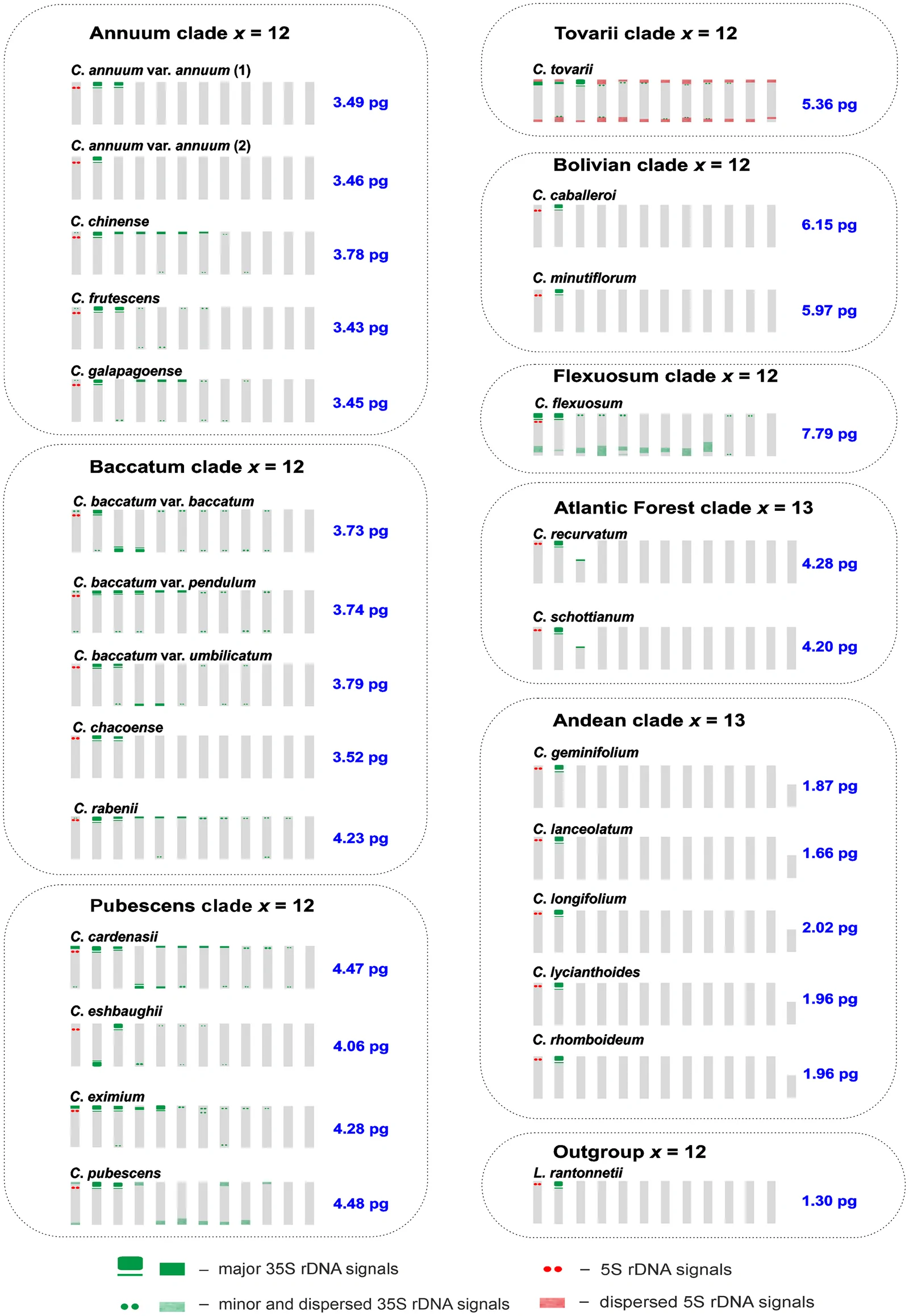
Number and localization of 35S (green) and 5S (red) rDNA loci in 24 *Capsicum* taxa and an outgroup, *Lycianthes rantonnetii*, using generalized ideograms. All chromosomes were depicted as uniformly sized, irrespective of their actual lengths, except for the smallest chromosome in the Andean and Atlantic Forest clades (both *x* = 13), which was clearly distinguishable to minimize the risk of data over-interpretation due to overall similarity of size and morphology of 12 chromosome pairs and frequent inability to unequivocally identify centromere position.

The numbers and distribution patterns of the 35S rDNA loci differed considerably between the species, from one in all analyzed species of the most basal Andean clade, *C. annuum* var. *annuum*, *C. caballeroi*, and *C. minutiflorum*, to 18 in *C. flexuosum*. Most loci were localized in distal regions of chromosomes, with only a few interstitial loci detected. The number of loci within the Annuum clade varied from either one or two in two different accessions of *C*. *annuum* var. *annuum* to eight in *C. frutescens* and 11 in *C*. *chinense* and *C. galapagoense* (**Figures 2A–E**). The number of major loci varied from one in *C. annuum* (**Figure 2B**) to six in *C. chinense* (**Figure 2C**). Loci number within the Baccatum clade ranged from two in *C*. *chacoense* to 16 in *C*. *baccatum* var. *baccatum* and var. *pendulum* (**Figures 2F–J**), with minor loci present in all species, but *C. chacoense* (**Figure 2I**). All four species of the Pubescens clade possessed both major (two to 9) and minor (six to 10) loci (**Figures 2K–N**). The loci classified as “minor” in cultivated *C. pubescens* were larger, but different in appearance from “minor” loci of the remaining crown group species, being distinctly dispersed/diffused (**Figure 2N**). *Capsicum tovarii* of the monotypic Tovarii clade had 14 loci (3 major) of 35S rDNA (**Figure 3A**). *Capsicum caballeroi* and *C*. *minutiflorum*, two analyzed species of the Bolivian clade, both had only one locus of 35S rDNA and no minor loci (**Figures 3B, C**). The sole member of the Flexuosum clade, *C. flexuosum*, possessed 18 loci, including two major and 16 “minor” distal and interstitial loci that were, in fact, larger in size, occupying up to one-fourth of the individual chromosome length, but appeared dispersed rather than compact (**Figure 3D**). Both Atlantic Forest clade species, *C. recurvatum* and *C*. *schottianum*, had two major and no minor loci (**Figures 3E, F**). All the members of the basal Andean clade (*C*. *geminifolium*, *C*. *lanceolatum*, *C*. *longifolium*, *C*. *lycianthoides*, and *C*. *rhomboideum*), as well as *L. rantonnetii* had only one major locus of 35S rDNA (**Figures 3G–L**). Pooled oligo probes representing selected and exclusively coding regions of 18S, 5.8S and 25S rDNAs were mapped in *C. flexuosum*, *C. galapagoense* and *C. geminifolium*, three species that varied in number and type of minor loci. The number and localization of detected major and minor loci corresponded to those detected using the larger 18S/25S rDNA probes (**Supplementary Figure 2**).

In this study, generalized ideograms were used to illustrate the number and distribution of 5S and 35S rDNA loci. All chromosomes were depicted as uniformly sized rectangles, irrespective of their actual lengths, except for the smallest (13^th^) chromosome in the Andean and Atlantic Forest clades (both *x* = 13), which was clearly distinguishable. This strategy minimized the risk of data over-interpretation that could result from: (1) overall size similarity of all 12 chromosome pairs and thus inability to unequivocally identify all homologous chromosome pairs, and (2) similar morphology of all chromosomes (meta- to submetacentric) and inability to unequivocally identify centromere position (**Figure 4**).

### 3.3 Ancestral state reconstructions of genome size, base chromosome number and rDNA loci numbers

The ancestral state of the base chromosome number for the genus was inferred as *x* = 12 using the phytools approach (**Figure 5A**). Consistent with the best-fitting model, two subsequent independent ascending dysploidy events were inferred, leading to the origin of *x* = 13 in the basal Andean clade and in the Atlantic Forest clade (**Figure 5A**).The reconstruction under the best-fitting Dys model (dysploidy-only) selected by ChromEvol also recovered *x* = 12 as the ancestral state of *Capsicum* and identified two independent transitions from *x* = 12 to *x* = 13, occurring at the same nodes as those reconstructed using phytools. No descending dysploidy events (*x* = 13 to *x* = 12) were detected (**Supplementary Data 4**).

**Figure 5:**
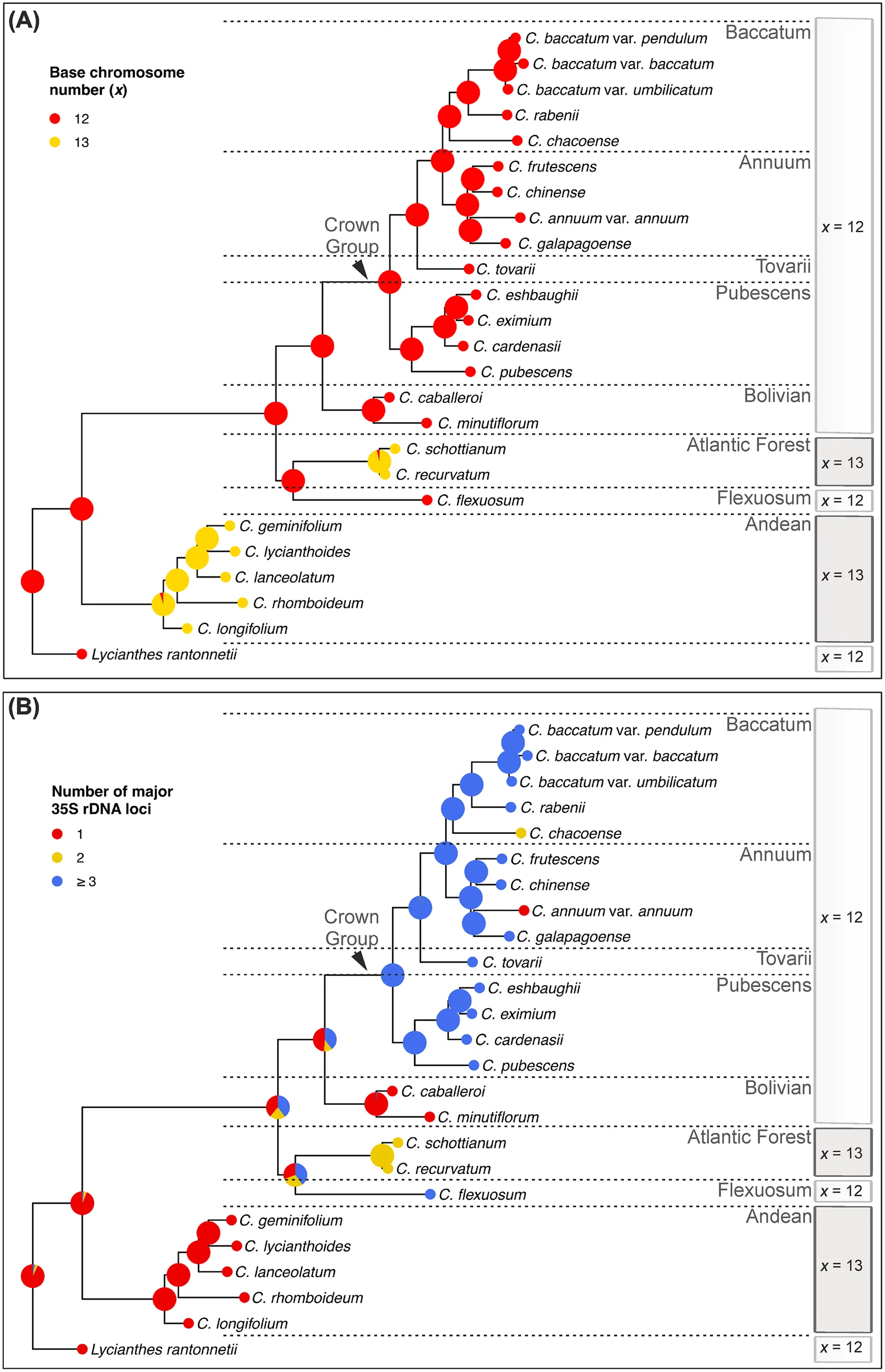
Ancestral state reconstructions of **(A)** the base chromosome number and **(B)** 35S rDNA loci numbers in the genus *Capsicum*. The recognized clades are indicated. The outgroup is represented by one species of the sister genus *Lycianthes*. In **(A)**, the proportion of state 12 in the MRCA of the Andean and Atlantic Forest clades is enlarged for clarity, as its actual value (0.005) falls below the figure’s visible resolution.

The ancestral state reconstruction of the major 35S rDNA loci number, performed under the best-fitting model ER, revealed a highly conserved evolutionary pattern across the genus (**Figure 5B**). The most recent common ancestor (MRCA) of *Capsicum* was reconstructed to have possessed a single locus, a condition that was retained throughout the diversification of the basal Andean clade. The reconstruction of the following diverging nodes, giving rise to the remaining extant clades, did not allow for unambiguous inference of any of the states. Within the lineage comprising the Flexuosum and Atlantic Forest clades, the MRCA exhibited high uncertainty, splitting into two distinct evolutionary paths: a transition to two loci in the Atlantic Forest species and an increase to three or more loci in *C. flexuosum*. The species of the Bolivian clade, *C. caballeroi* and *C. minutiflorum*, retained the single-locus state, which was inferred as a direct retention of the basal ancestral number of the genus rather than a subsequent reduction. Another independent transition to three or more loci was inferred for the MRCA of the crown group. This state dominates across the crown clades Pubescens, Tovarii, Baccatum, and Annuum, with only two homoplastic reversals detected: a reversal to two loci in *C. chacoense* and a single reduction event to one locus in *C. annuum* var. *annuum*. Within the species bearing three or more loci, two distinct patterns were observed. The majority of species predominantly exhibited distal major and minor loci that varied in size (with the smallest ones often at the detection limit) but shared a similar signal density. In contrast, *C. flexuosum* and the crown group species *C. pubescens* displayed two distinct types of loci distinguishable in most chromosomal spreads: two dense, compact, and likely active loci, with secondary constrictions occasionally clearly delineated, and the remaining larger, distal or interstitial blocks that appeared characteristically dispersed. Consequently, although there are two distinct categories of loci, for methodological consistency both species were classified as carrying “three or more” loci.

The genome size for the MRCA of *Capsicum* was reconstructed as relatively small (2.31 pg; **Figure 6**). This ancestral state was retained or further reduced exclusively in the most basal Andean lineage. All other derived lineages experienced genome upsizing (5.02 pg in the MRCA of all these clades) with several independent subsequent downsizing events. One such reduction occurred at the origin of the whole crown group (clades Annuum, Pubescens, Baccatum and Tovarii; 4.63 pg), with subsequent further reduction in the Annuum and Baccatum clades (4.02 pg inferred in their MRCA), both of which encompassed four of five domesticated species (**Figure 6**). Genome upsizing was also inferred for the MRCA of the Flexuosum and Atlantic Forest clades (5.18 pg), whereas the subsequent speciation and diversification of the latter was accompanied by genome size reduction (**Figure 6**). The most significant and pronounced genome size increase was observed in the monotypic Flexuosum clade, with *C. flexuosum* possessing the largest genome size in the whole genus.

**Figure 6:**
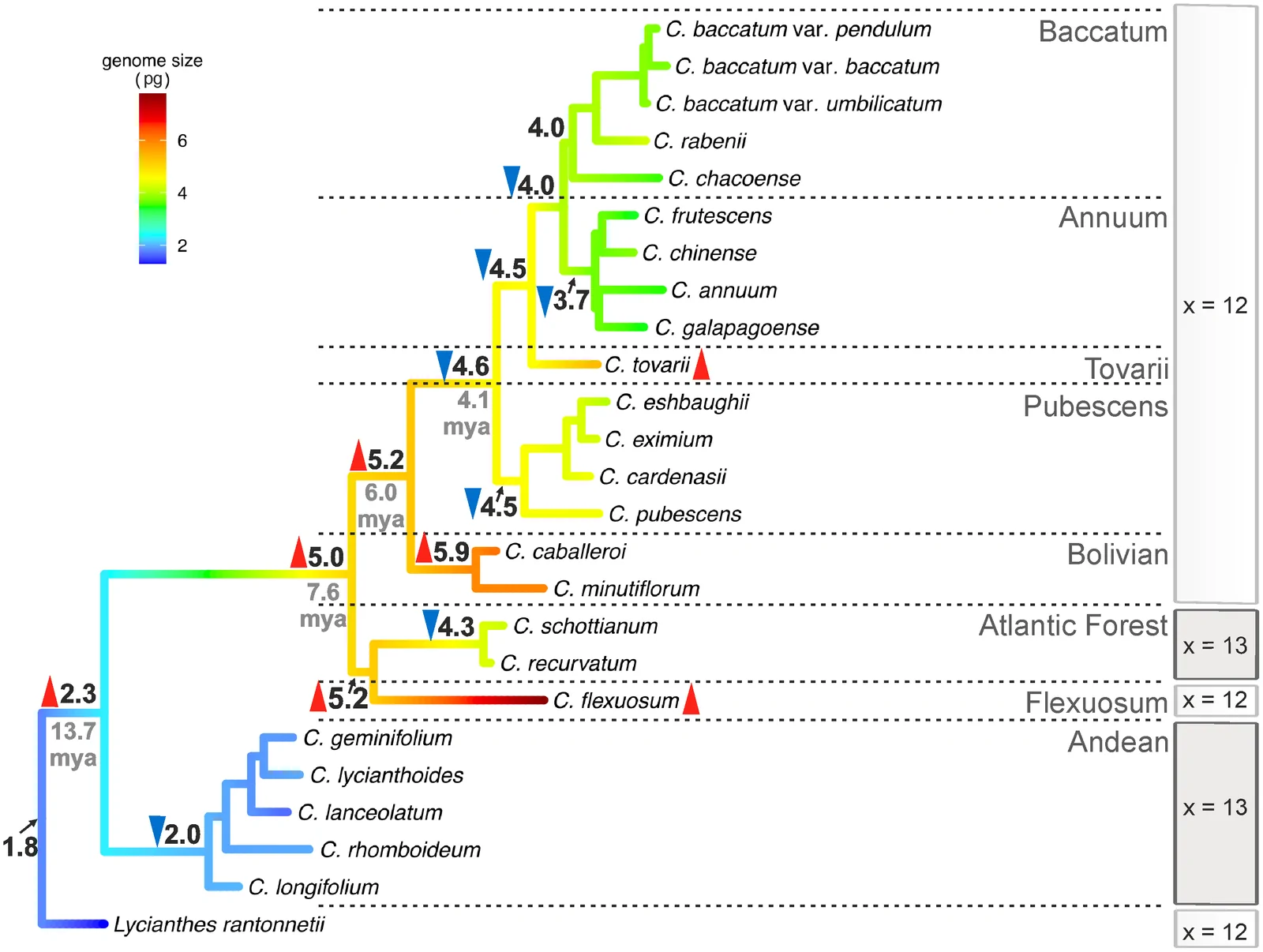
Ancestral state reconstruction of the genome size in the genus *Capsicum*. Genome sizes inferred for major nodes are indicated in black. Age of the major nodes indicated in grey below the nodes (mya- million years ago; Carrizo García et al., 2022). Blue arrowheads indicate decreases and red arrowheads indicate increases of the genome sizes at the major nodes. The recognized clades are indicated. The outgroup is represented by one species of the sister genus *Lycianthes*.

All three analyzed characters carried phylogenetic signal (**Supplementary Data 3**). The genome size showed strong signal, with both Pagel’s λ (0.999927, p-value = 3.03 × 10^−^¹²) and Blomberg’s K (K = 2.47196, p-value = 0.001) indicating that closely related species tend to share similar genome sizes, with the high K value (> 1) suggesting even greater similarity among close relatives than expected under a simple Brownian motion model. For the binary character, base chromosome number (*x*), the estimated D statistic (-0.894) was significantly different from a random phylogenetic structure (p-value < 0.001) and not significantly different from a Brownian motion model (p-value = 0.906), both indicating a highly conserved and non-random evolutionary pattern. Following the PGLMM, a strong phylogenetic signal was revealed for both discrete traits. The base chromosome number was highly conserved across the phylogeny (H^2^ mode = 0.999, 95% HPD = 0.998-0.999), and a similarly conserved trend was found for the major 35S rDNA loci (H^2^ mode = 0.997, 95% HPD = 0.900-0.999; **Supplementary Data 3**).

## 4 Discussion

The genus *Capsicum*, with a relatively moderate number of species and lack of polyploidy in its evolutionary history, offers an excellent system in which to analyse genome composition, patterns and mechanisms of the evolution of genome size, as well as structure and evolution of the karyotypes. Novel data reported in this study allowed for more comprehensive analyses of the evolution of genome size, chromosome numbers, as well as 35S rDNA loci numbers and localization over past nearly 14 million years of the evolution of chile peppers (Carrizo García et al., 2022).

Nearly 5-fold range of genome sizes with the smallest values in the Andean clade and the largest in the monotypic Flexuosum clade, in the absence of polyploidy, implies dynamic changes in repeatome composition accompanying diversification of the lineages. Ancestral state reconstruction inferred a low ancestral genome size for the whole genus (c. 2 pg), a trait that has been retained, and even accompanied by slight genome downsizing in some of the members of the basal Andean clade. The evolution and diversification of the remaining lineages were accompanied by both increases and a few subsequent reductions of genome sizes. Most significant increases affected *C. flexuosum*, *C. tovarii* and the species of the Bolivian clade. In contrast, the derived clades of the crown group, most of which comprised domesticated species, experienced nearly exclusively reductions of genome sizes. This contrasts with derived karyotype and genome size traits previously postulated for chile peppers, specifically increases in karyotype length and asymmetry, nuclear DNA amount and heterochromatin content (Moscone et al., 2007). Based on those basic and descriptive characters, *C. chacoense* was suggested to be the most primitive taxon of the genus, and the Atlantic Forest species the most derived ones (Moscone et al., 2007). This hypothesis could not be confidently corroborated by the patterns inferred from phylogenetic analyses using RADseq data (Carrizo García et al., 2022) which suggested the Andean clade to be the most basal and crown group taxa to be most derived. Inferences of the patterns of genome size evolution revealed dynamic upsizing (Flexuosum, Bolivian and Tovarii clades) and downsizing (all remaining derived clades) that has occurred independently in different lineages and clades, and no directionality, in contrast to previous hypotheses based on chromosomal data (Moscone et al., 2007). Comparative genus-wide repeatome analyses will shed more light into the dynamics and major players involved in genome expansion and contraction in individual lineages of *Capsicum* (Novák et al., 2014; McCann et al., 2020).

Beyond genome size, karyotype architecture itself differs across lineages, most notably in the base chromosome number. All chile pepper species possess rather uniform symmetrical karyotypes with mostly metacentric and submetacentric chromosomes, similarly to other related Solanaceae genera (Wu and Tanksley, 2010), except for the occurrence of an additional subtelocentric chromosome in two *x* = 13 *Capsicum* lineages (Scaldaferro et al., 2016; Scaldaferro and Moscone, 2019). The presence of two base chromosome numbers, *x* = 12 and 13, in *Capsicum* has been inferred to be uniform within each of the nine distinct clades but differed among the clades. The base chromosome number of *x* = 12, found also in the closest sister genus, *Lycianthes*, has been inferred in the current study as ancestral for the whole *Capsicum* genus with two independent ascending dysploidy events leading to a new base chromosome number of *x* = 13. One such event has occurred in the MRCA of the most basal and oldest Andean clade and the second in the MRCA of the derived and younger Atlantic Forest clade (4.5 myo and 3 myo, respectively; Carrizo García et al., 2022). The presence of the base number of *x* = 13 in the most basal and oldest Andean clade as well as the distinct morphology of the 13^th^ smallest chromosome shared between both *x* = 13 clades led to long standing dispute over the ancestral base chromosome of the genus (Pozzobon et al., 2006; Moscone et al., 2007). Arguments supporting *x* = 12 as the ancestral base chromosome number have largely remained speculative, including, for example, the reported absence of telomeric sequences at ectopic locations in species with 2*n* = 24 (Moscone et al., 2007). Conversely, the significant differences in morphological and ecological characteristics of the Atlantic Forest and Andean clades supported the hypothesis that the native south-eastern Brazilian species (*x* = 13) belonged to the ancestral *Capsicum* gene pool. The lack of robust molecular phylogenetic hypotheses did not allow for rigorous testing of the hypotheses of single vs. independent origins of the two clades carrying *x* = 13 chromosomes (Moscone et al., 2007). Ancestral state reconstructions using RADseq based phylogenetic framework presented in this study not only supported independent origin of *x* = 13 as the derived character for the genus but permitted also the conclusion that these two events have been accompanied by significantly different evolution of the genome sizes. Whereas Andean clade species experienced genome downsizing, the Atlantic Forest clade species upsized their genomes. Karyotype restructuring, leading to independent base chromosome number change from *x* = 12 to *x* = 13 raises also the question of homoeology between the smallest subtelocentric 13^th^ chromosomes present in these two lineages, a character that has previously been put forward in support of the shared origin of *x* = 13 lineages. Comparative chromosome painting, together with whole-genome assemblies and chromosome-level genomic analyses, may help elucidate the genomic origin and structural basis of these independently derived small chromosomes and clarify the mechanisms underlying their formation.

In the absence of polyploidy, with only a few reports of polyploid individuals in cultivated species that can be attributed to human-assisted selection (Jha et al., 2017; Scaldaferro and Moscone, 2019; Barboza et al., 2022), 5-fold genome size variation in chile peppers must stem from dynamic evolution of repetitive DNA types (Bennetzen and Wang, 2014; He et al., 2024). Ty3/*gypsy* retrotransposons have been inferred to contribute to most of the genome size increases in several analyzed species of *Capsicum* (Park et al., 2012; de Assis et al., 2020; Chen et al., 2024; Liu et al., 2025). One of the repeat types that also contributes to the dynamics of genome evolution is rDNA. The conservative approach to inferring ancestral character states for the number of rDNA loci used in this study is intended to capture broad patterns of 35S rDNA locus-number evolution without making assumptions about locus homology or activity, providing a basic framework that can be further refined and tested using genomic data. The numbers of 35S rDNA loci, or more specifically, rDNA signals, in *Capsicum* species reported both previously and in the current study are largely consistent for species that carry a single locus (Andean clade; Scaldaferro and Barboza, 2023) but differ in most of the species of the crown group, all of which possess numerous minor loci. These small loci, which should perhaps more correctly be referred to as regions that hybridize to 35S rDNA probe, differ in number not only among studies, but also among accessions and individuals (“cytotypes”; Scaldaferro and Moscone, 2019) and even preparations analyzed (current study), most of them being at the limit of detection. One to four active rDNA loci per haploid genome have previously been reported in *Capsicum* using silver staining, implying the presence of maximum four 35S rDNA loci in the analyzed species (Scaldaferro et al., 2016), with the remaining minor signals likely representing either inactive loci, DNA motifs derived from rDNA monomers (e.g., IGS-derived satellite DNAs) or perhaps massively amplified truncated rDNA monomers. This is also largely supported by the reported numbers of active rDNA loci versus the total numbers of detected rDNA hybridization signals. Atlantic Forest and Caatinga clade species were reported to possess either one or two active rDNA loci, *C. flexuosum* two, *C. tovarii* two or three, and the species of the Pubescens, Baccatum and Andean clades one to three active loci (Scaldaferro and Moscone, 2019).

35S rDNA monomers are known to have given rise to satellite DNAs in several angiosperm lineages (Jo et al., 2011; Almeida et al., 2012; Sader et al., 2021), mostly, but not only, via amplification of DNA motifs from within IGS (Intergenic Spacer) region. Multiple small minor loci in four *Capsicum* clades, as well as the larger diffuse blocks of chromosomes hybridizing to 35S rDNA probe in *C. flexuosum* and *C. pubescens* might also represent, at least partly, rDNA-derived tandem repeats or fragmented and truncated rDNA monomers, perhaps interspersed with other tandem repeats or autonomous or truncated retrotransposons (de Assis et al., 2020, 2023). Two previous studies that have specifically addressed this question in cultivated *Capsicum* species provided contrasting and partly incongruent results (Jo et al., 2011; Grabiele et al., 2018). One of these studies inferred the presence of satellite DNA resulting from independent amplification of either whole or partial IGS region (Jo et al., 2011). However, the following study (Grabiele et al., 2018) failed to verify these results and instead presented evidence of the independent amplification of larger fragments of 35S rDNA monomers spanning not only IGS region but also parts of both larger coding regions (especially 25S rDNA) and internal spacers. Amplification of these regions was suggested to have given rise to a large “mega-satellite” rDNA-derived family that is now found amplified in distal regions of many chromosomes (Grabiele et al., 2018). The probes used in the current study represented larger 18S or 25S rDNA coding regions (of *Arabidopsis thaliana* origin; Gruendler et al., 1991) which may partially contain small regions of the ITS/IGS. Thus, oligo probes spanning selected and exclusively coding regions of 18S, 5.8S and 25S rDNAs were designed and used for additional mapping in chromosomes. These pooled oligo probes, however, also hybridized to the same chromosomal regions as *Arabidopsis thaliana* 18S and 25S rDNA probes, supporting rather the hypothesis of the presence of “mega-satellite” of rDNA origin (Grabiele et al., 2018) than IGS-derived satellite DNA (Jo et al., 2011) or larger blocks of truncated genic regions. The presence of loci of satellite DNAs that lack any sequence similarity to rDNA detected in distal regions of many *Capsicum* chromosomes (de Assis et al., 2023), adjacent to or co-localizing with rDNA hybridization signals, suggests even more complex organization of those chromosomal regions.

Distinct appearance of some 35S rDNA signals detected in *C. flexuosum* and *C. pubescens* suggested that these additional large and dispersed blocks that hybridize to 35S rDNA probes might represent tandem repeats that have originated independently in these two unrelated species. Analyses of IGS of *C. pubescens* revealed the presence of distinct motif not found in other species, that might have contributed - via independent amplification - to the origin of novel and distinct rDNA-derived tandem repeat (Grabiele et al., 2018). This implies that the dynamic of evolution of ribosomal genes in the genus *Capsicum* may be more complex, perhaps resulting from recurrent and independent amplification(s) of various segments of 35S rDNA monomers. Despite the availability of T2T (telomere to telomere) genome assemblies of several *Capsicum* species, the composition, dynamics and role of satellitome in genome size and chromosome structure evolution of peppers remains largely unknown (Liu et al., 2023; Chen et al., 2024; Zhang et al., 2025), with only a few satellite DNAs identified in *C. annuum* and other cultivated species so far, none of which had any DNA sequence similarity to any of the rDNAs (de Assis et al., 2023). Detailed analyses of whole genome assemblies of multiple *Capsicum* genomes might be able to shed more light into the arrangement and evolution of rDNA-related chromosomal regions and their potential contribution to the satellitomes (Zhang et al., 2025).

In contrast to high levels of variation of 35S rDNA signals, some representing active rDNA loci, most of the chile pepper species possessed single locus of 5S rDNA. Only two exceptions to that pattern were found, the uniquely high number of large distal regions of all chromosomes hybridizing to 5S rDNA in the accession of *C. tovarii* analyzed in the present study and the presence of three loci in *C. regale* (Scaldaferro et al., 2016; Scaldaferro and Barboza, 2023). Large number of mostly dispersed 5S rDNA-hybridizing blocks in *C. tovarii* reported in the current study contrasts with single locus reported for another *C. tovarii* accession, but is in line with multiple heterochromatic blocks reported in yet another *C. tovarii* accession (Scaldaferro and Moscone, 2019). The presence of so many heterochromatic blocks that hybridize to 5S rDNA genic probe may suggest the presence of satellite DNA originating from within 5S rDNA monomer in at least some populations/cytotypes of *C. tovarii*. Comparative analyses of 5S rDNA monomers in several *Capsicum* species allowed for the identification of a unique and large insertion (123 bp) in the structural region I of the rDNA NTS (non-transcribed spacer) of *C. tovarii* that was absent in all other chile pepper taxa (Grabiele et al., 2019). This insertion, present in some, but not all, of the sequenced monomers of 5S rDNA contained a repeated region at 5’ and 3’ associated with a region similar to 5S rDNA gene (similarity c. 50%), which was hypothesized to have originated by unequal crossing over within the coding ribosomal region, making this NTS the longest within the genus (Grabiele et al., 2019). The probe representing exclusively 5S rDNA coding region used in this study detected not only the active 5S rDNA locus/loci, but also to the modified and highly amplified regions/blocks, albeit to a lesser extent. Such cross hybridization is congruent with previously reported similarity of *C. tovarii*-specific motif found it its NTS region to the gene itself. Whether the modified monomers of 5S rDNA containing the unique motif are non-functional or fragmented and whether they are organized in unique loci or interspersed with regular and fully functional 5S rDNA monomers or perhaps also other repeats, remains to be analyzed. The presence of three 5S rDNA loci in *C. regale* of the Andean lineage (Scaldaferro and Barboza, 2023) might also suggest the presence of novel tandem repeats. Two of the three loci were reported to have appeared as larger blocks and were suggested to represent putative non-coding repeats of 5S rDNA origin (Scaldaferro and Barboza, 2023). If indeed true, it provides further evidence for high levels of dynamics of rDNA evolution in chile pepper genomes.

This study provides the first integrated analysis of extant character states and their ancestral reconstruction for genome size, base chromosome number, and rDNA loci number within a phylogenetic framework for *Capsicum*, based on data collected from the same individuals across 22 species and three varieties. The MRCA of *Capsicum* was reconstructed as having a small genome (~2.3 pg/1C), *x* = 12 chromosomes and a single 35S rDNA locus. Major genomic transitions, including shifts in genome size, ascending dysploidy events (*x* = 12 → 13), and 35S rDNA expansions, coincided primarily with the divergence of major clades rather than intra-clade speciation, resulting in strong genomic uniformity within lineages. Furthermore, ancestral state reconstructions indicate that patterns and dynamics of genome size and base chromosome number evolution differ across *Capsicum*, as the two independent ascending dysploidy events in the Andean and Atlantic Forest clades were accompanied by opposing genome size trajectories. In the complete absence of polyploidy, the nearly 5-fold variation in genome size observed across the genus may stem predominantly from dynamic changes in repetitive DNA content.

The ancestral state reconstructions provide a robust framework to address mechanisms involved in the evolution of these characters across this exclusively diploid genus. Analyses of the composition of the repetitive DNA fractions will clarify the contributions of various repeat lineages to the variation of genome sizes. In particular, the extent and type of contributions of both 5S and 35S ribosomal DNA units to the origin of novel satellite DNAs can be addressed. Finally, comparative analyses of chromosome-level whole-genome assemblies representing all major *Capsicum* lineages should allow for ultimate testing of the hypothesis that the two ascending dysploid lineages with a base chromosome number of *x* = 13 evolved independently.

## Data Availability

The original contributions presented in the study are included in the article/**Supplementary Material**. Further inquiries can be directed to the corresponding author.
