# Supplementary material for: Patterns of base chromosome numbers, genome size and rDNA loci evolution in the genus Capsicum (Solanaceae)
Source: Front Plant Sci. 2026 Sep 28;17:1959260. doi: 10.3389/fpls.2026.1959260 (PMC13655049; doi:10.3389/fpls.2026.1959260)
Supplement: Supplementary file 1 [file DataSheet1.pdf]

Supplementary Material - Figures

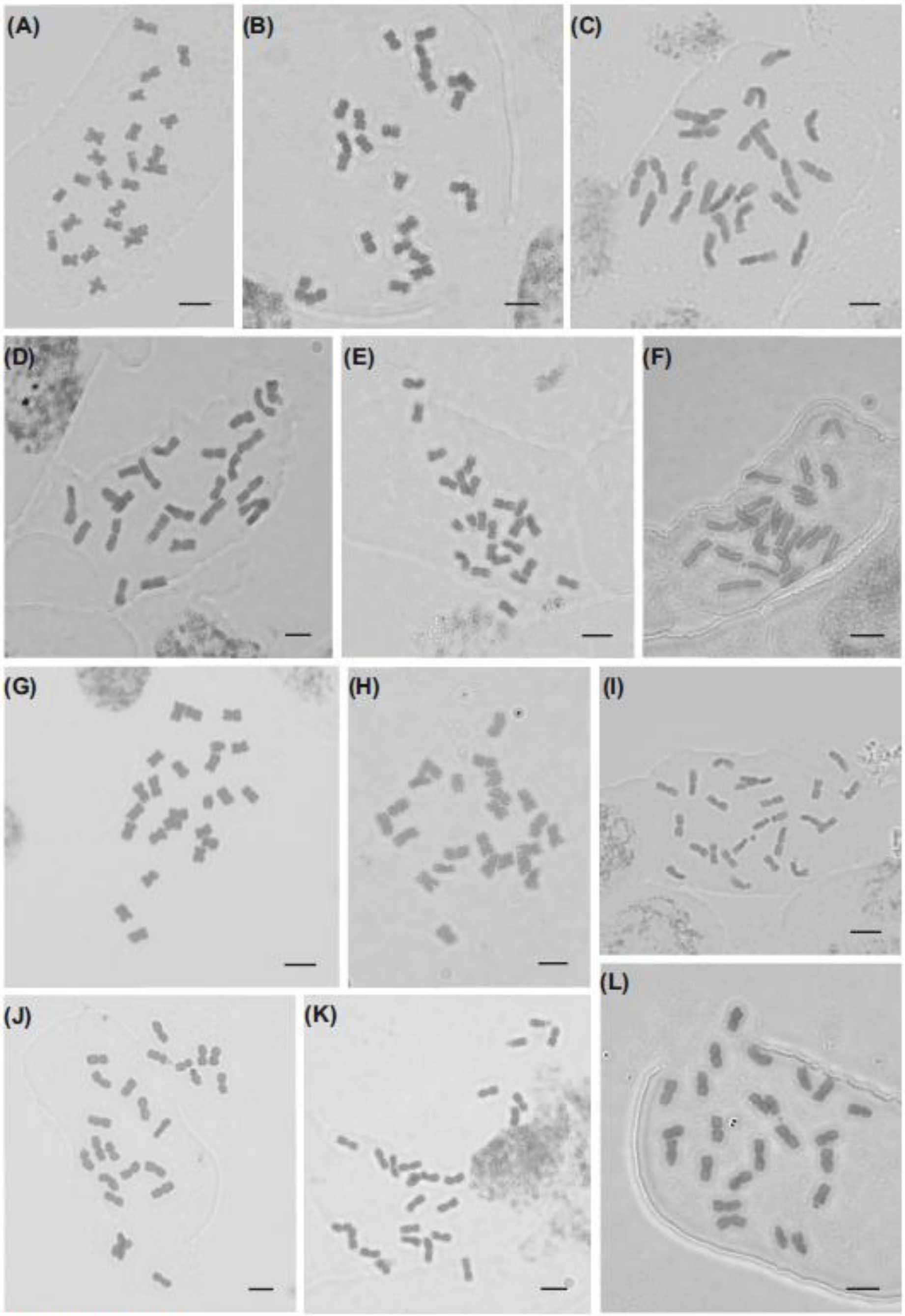

Supplementary Figure 1.

Metaphase mitotic chromosomes of analyzed *Capsicum* species. **A-E Annuum clade**, 2*n* = 24: (A) *C. annuum* var. *annuum* (1), (B) *C. annuum* var. *annuum* (2), (C) *C. chinense*, (D) *C. frutescens*, (E) *C. galapagoense*. **F-J Baccatum clade**, 2*n* = 24: (F) *C. baccatum* var. *baccatum*, (G) *C. baccatum* var. *pendulum*, (H) *C. baccatum* var. *umbilicatum*, (I) *C. chacoense*, (J) *C. rabenii*. **K-N Pubescens clade**, 2*n* = 24: (K) *C. cardenasii*, (L) *C. eshbaughii*, (M) *C. eximium*, (N) *C. pubescens*. **O Tovarii clade**, 2*n* = 24: (O) *C. tovarii*. **P-Q Bolivian clade**, 2*n* = 24: (P) *C. caballeroi*, (Q) *C. minutiflorum*. **R Flexuosum clade**, 2*n* = 24: (R) *C. flexuosum*. **S-T Atlantic Forest clade**, 2*n* = 26: (S) *C. recurvatum*, (T) *C. schottianum*. **U-Y Andean clade**, 2*n* = 26: (U) *C. geminifolium*, (V) *C. lanceolatum*, (W) *C. longifolium*, (X) *C. lycianthoides*, (Y) *C. rhomboideum*. **Z Outgroup**, 2*n* = 24: (Z) *Lycianthes rantonnetii*. Scale bar, 5 µm.

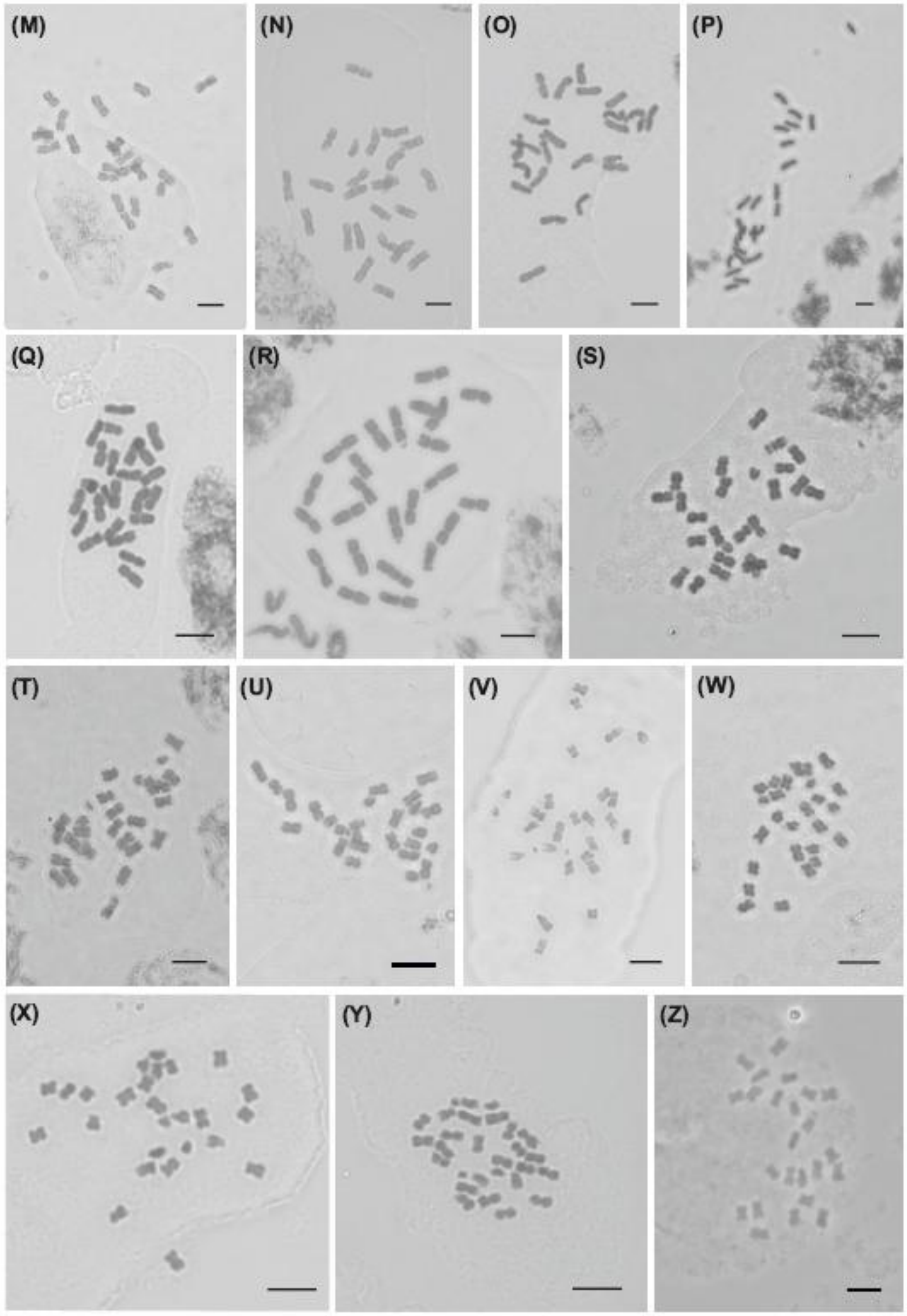

Supplementary Figure 1 - contd.
Metaphase mitotic chromosomes of analyzed *Capsicum* species. **A-E Annuum clade**, 2*n* = 24: (A) *C. annuum* var. *annuum* (1), (B) *C. annuum* var. *annuum* (2), (C) *C. chinense*, (D) *C. frutescens*, (E) *C. galapagoense*. **F-J Baccatum clade**, 2*n* = 24: (F) *C. baccatum* var. *baccatum*, (G) *C. baccatum* var. *pendulum*, (H) *C. baccatum* var. *umbilicatum*, (I) *C. chacoense*, (J) *C. rabenii*. **K-N Pubescens clade**, 2*n* = 24: (K) *C. cardenasii*, (L) *C. eshbaughii*, (M) *C. eximium*, (N) *C. pubescens*. **O Tovarii clade**, 2*n* = 24: (O) *C. tovarii*. **P-Q Bolivian clade**, 2*n* = 24: (P) *C. caballeroi*, (Q) *C. minutiflorum*. **R Flexuosum clade**, 2*n* = 24: (R) *C. flexuosum*. **S-T Atlantic Forest clade**, 2*n* = 26: (S) *C. recurvatum*, (T) *C. schottianum*. **U-Y Andean clade**, 2*n* = 26: (U) *C. geminifolium*, (V) *C. lanceolatum*, (W) *C. longifolium*, (X) *C. lycianthoides*, (Y) *C. rhomboideum*. **Z Outgroup**, 2*n* = 24: (Z) *Lycianthes rantonnetii*. Scale bar, 5 μm.

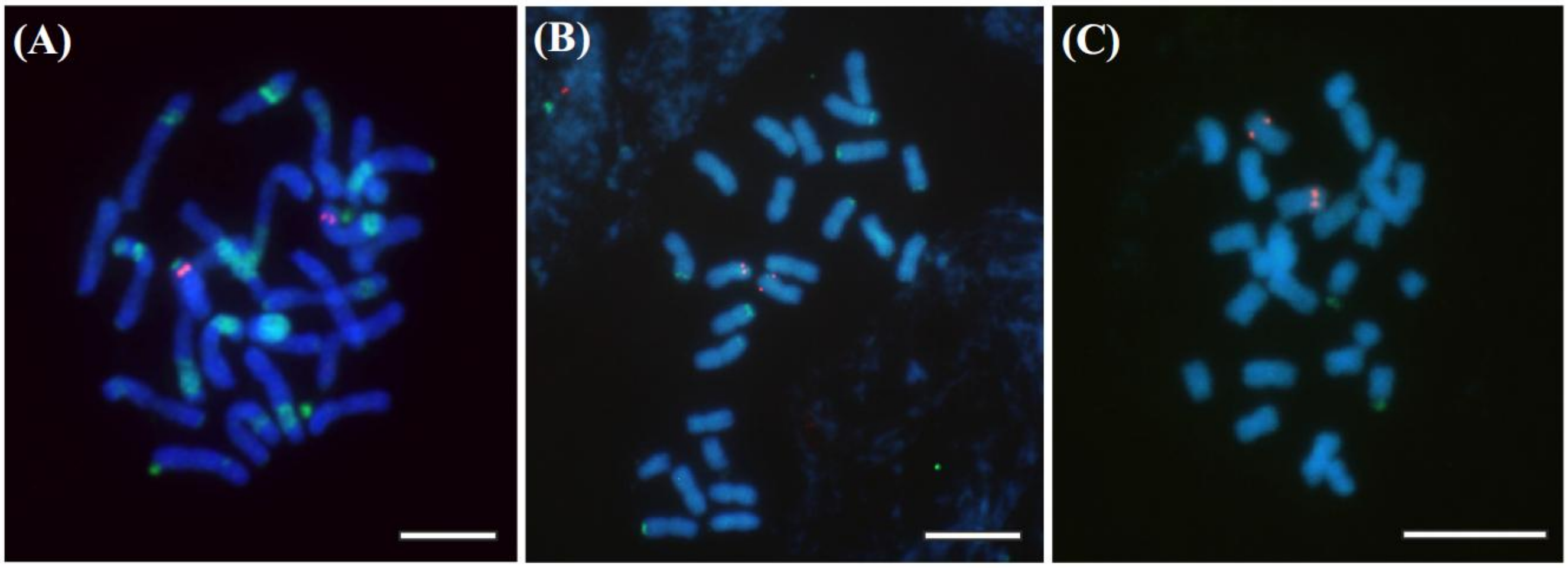

Supplementary Figure 2.
Localization of 35S rDNA (pooled oligo probes for 18S, 5.8 and 25S rRNA genes; green) and 5S rDNA (5S rRNA gene; red) in mitotic metaphase chromosomes of three selected *Capsicum* species. (A) *Capsicum flexuosum*; (B) *Capsicum galapagoense*; (C) *Capsicum geminifolium*. Scale bar, 5 µm.
