# Supplementary material for: Patterns of base chromosome numbers, genome size and rDNA loci evolution in the genus Capsicum (Solanaceae)
Source: Front Plant Sci. 2026 Sep 28;17:1959260. doi: 10.3389/fpls.2026.1959260 (PMC13655049; doi:10.3389/fpls.2026.1959260)
Supplement: Supplementary file 2 [file DataSheet2.pdf]

Supplementary Data - Tables

Supplementary Table 1. Origin of seeds and voucher list for the analyzed species.

Supplementary Table 2. Chromosome numbers, ribosomal DNA loci (5S and 35S rDNA) numbers and genome sizes (1C values in pg) reported previously for the genus *Capsicum*.

Supplementary Table 1. Origin of seeds and voucher list for the analyzed species. Abbreviations: HBV – Hortus Botanicus Vindobonensis (Vienna Botanical Garden), Austria; WU – Herbarium of the University of Vienna, Austria; CORD – Botanical Museum of Córdoba, National University of Córdoba, Argentina. Each analyzed taxon was represented by a single individual.

Taxon	Origin of seeds (country, locality)	Voucher
**Annuum clade**		
C. annuum L. var. *annuum*	fruits acquired at marketplace	HBV, live collection
C. annuum L. var. *annuum*	fruits acquired at marketplace	HBV, live collection
C. chinense Jacq.	Brazil: Rio de Janeiro (fruits acquired at market place)	HBV, live collection
C. frutescens L.	Brazil: São Paulo (fruits acquired at marketplace)	HBV, live collection
C. galapagoense Hunz.	Netherlands: Centre for Genetic Resources (CGN 22208)	Carrizo García 99, WU
**Baccatum clade**		
C. baccatum L. var. *baccatum*	Argentina: Salta, Salta	Carrizo García 95, WU
C. baccatum var. *pendulum* (Willd.) Eshbaugh	Bolivia: Santa Cruz de la Sierra (fruits acquired at marketplace)	Carrizo García 96, WU
C. baccatum var. *umbilicatum* (Vell.) Hunz. & Barboza	Argentina: Córdoba (fruits acquired at marketplace)	HBV, live collection
C. chacoense Hunz.	Argentina: Córdoba, Panaholma	Carrizo García & Fernández 90, CORD
C. rabenii Sendtn.	Netherlands: Centre for Genetic Resources (CGN 22795)	Carrizo García 86, WU
**Pubescens clade**		
C. cardenasii Heiser & P.G.Sm.	Bolivia: La Paz (fruits acquired at marketplace)	Carrizo García 76, WU
C. eshbaughii Barboza	Bolivia: Santa Cruz, Samaipata	Carrizo García & Fernández 67, CORD
C. eximium Hunz.	Argentina: Jujuy, El Fuerte	Carrizo García & Fernández 53, CORD
C. pubescens Ruiz & Pav.	Bolivia: La Paz, Chulumani, Apa Apa	Carrizo García 75, WU
**Tovarii clade**		
C. tovarii Eshbaugh, P.G.Sm.& Nickerent	Netherlands: Centre for Genetic Resources (CGN PI 606708/ NMCA 90008)	Carrizo García 74, CORD
**Bolivian clade**		
C. caballeroi M.Nee	Bolivia: Santa Cruz, El Empalme	Barboza 4907, CORD
C. minutiflorum (Rusby) Hunz.	Bolivia, Santa Cruz, Cuevas (donated by A.A. Cocucci)	Carrizo García 77, WU
**Flexuosum clade**		
C. flexuosum Sendtn.	Argentina: Misiones, Candelaria	Daviña & Hofni 599, CORD
**Atlantic Forest clade**		
C. recurvatum Witasek	Brazil: Paraná, Morretes	Barboza 1632, CORD
C. schottianum Sendtn.	Brazil: São Paulo, Paranapiacaba	Barboza 3635, CORD
**Andean clade**		

C. geminifolium Barboza & Leiva	Colombia: Caquetá	Orejuela et al. 2688, CORD
C. lanceolatum (Greenm.) C.V.Morton & Standl.	Spain: La Palma Co.	Carrizo García 73, WU
C. longifolium Barboza & Leiva	Ecuador: Zamora-Chinchipe, E. Científica San Francisco	Barboza & Leiva 4851, CORD
C. lycianthoides (Dammer) Hunz.	Colombia: Quindío, Circasia	Barboza & Beltrán 85, CORD
C. rhomboideum (Dunal) Kuntze	Venezuela: Táchira, San Cristóbal (donated by Y. García)	Carrizo García 97, WU
**Outgroup**		
L. rantonnetii (Carrière) Bitter	Argentina: Cordoba	CORD, live collection

Supplementary Table 2. Chromosome numbers, ribosomal DNA loci (5S and 35S rDNA) numbers and genome sizes (1C values in pg) reported previously for the genus *Capsicum*.

Taxon	*n*	2*n*	rDNA loci number		Genome size 1C (pg)
			35S	5S	
**Annuum clade**					
C. annuum var. *annuum*	-	24[1]	1[6]; 2[3]; 3[5,6,7,9]; 4[5,9]	1[3,4,5,6,7,8,9]	3.83[10]; 3.41[11]; 3.38[11]; 3.32[11]
C. annuum var. *glabriusculum*	-	24[1]	1[7,9]; 2[7,9]; 3[7,9]; 4[7,9]; 5[7,9]; 6[7,9]	1[7,9]	3.66[12]
C. chinense	12[1]	24[1]	2[6]; 4[3]; 5[7,9]	1[3,4,6,7,8,9]	4.02[10]; 3.41[11]; 3.43[11]
C. frutescens	-	24[1]	1[6]; 2[6]; 9[3,7,9]	1[3,4,6,7,8,9]	3.97[10]; 3.40[11]
C. galapagoense	12[1]	24[1]	-	-	-
**Baccatum clade**					
C. baccatum var. *baccatum*	-	24[1]	4[6]; 14[3]; 15[7,9]	1[3,4,6,7,9]	4.22[10]; 3.71[11]
C. baccatum var. *pendulum*	-	24[1]	14[7,9]	1[7,8,9]	4.20[10]; 3.71[11]; 3.68[11]
C. baccatum var. *umbilicatum*	-	24[1]	13[9]; 15[5]	1[5,9]	3.76[11]
C. chacoense	12[1]	24[1]	2[6]; 4[5,9]	1[5,6,8,9]	3.83[10]; 3.34[11]; 3.35[11]
C. rabenii	12[1]	24[1]	11-13[7,9]	1[7,8,9]	4.57[10]
**Pubescens clade**					
C. cardenasii	12[1]	24[1]	8-11[7,9]; 18[5,9]	1[5,7,8,9]	4.49[10]
C. eshbaughii	-	24[1]	-	-	-
C. eximium	12[1]	24[1]	6[7,9]	1[7,8,9]	4.35[10]; 4.06[11]
C. pubescens	-	24[1]	12[3]; 14[5,9]	1[3,4,5,8,9]	4.86[10]; 4.47[11]
**Tovarii clade**					
C. tovarii	12[1]	24[1]	8[7,9]	1[7,9]	3.97[10]
**Bolivian clade**					
C. caballeroi	-	24[1]	-	-	-
C. ceratocalyx	-	-	-	-	-
C. coccineum	-	-	-	-	-
C. minutiflorum	-	-	-	-	-
C. neei	-	-	-	-	-
**Flexuosum clade**					
C. flexuosum	12[1]	24[1]	14-15[7,9]	1[7,8,9]	7.2[12]
**Caatinga clade**					
C. caatingae	12[1]	24[1]	-	-	5.77[11]
C. longidentatum	-	24[1]	-	-	-
C. parvifolium	-	24[1]	-	-	-
**Atlantic Forest clade**					
C. campylopodium	13[1]	26[1]	-	-	4.53[11]; 5.74[11]
C. carassense	-	-	-	-	-
C. cornutum	13[1]	26[1]	-	-	-
C. friburgense	13[1]	-	-	-	-
C. hunzikerianum	-	26[1]	-	-	-
C. mirabile	13[1]	26[1]	-	-	-
C. mirum	-	-	-	-	-
C. muticum	-	-	-	-	-
C. pereirae	13[1]	26[1]	-	-	-
C. recurvatum	13[1]	26[1]	4[7,9]	1[7,9]	-
C. schottianum	13[1]	26[1]	-	-	-
C. villosum	13[1]	26[1]	30[7,9]	1[7,9]	-
**Andean clade**					
C. dimorphum	-	26[2]	1[2]	1[2]	-
C. geminifolium	-	26[2]	1[2]	1[2]	-
C. hookerianum	-	26[2]	1[2]	1[2]	-

C. lanceolatum	13[1]	26[2]	1[2]	1[2]	-
C. longifolium	-	26[1,2]	1[2]	1[2]	-
C. lycianthoides	-	26[1,2]	1[2]	1[2]	-
C. piuranum	-	26[1,2]	1[2]	1[2]	-
C. regale	-	26[1,2]	1[2]	3[2]	-
C. rhomboideum	-	26[1,2]	1[2,7,9]	1[2,7,8,9]	2.08[12]
**Unassigned clade**					
C. benoistii	-	-	-	-	-

[1] Barboza, G. E., Carrizo García, C., Bianchetti, L. B., Romero, M. V., and Scaldaferro, M. (2022). 'Monograph of wild and cultivated chili peppers (*Capsicum* L., Solanaceae). *PhytoKeys* 200, 1–423. doi.org/10.3897/phytokeys.200.71667 and references therein

[2] Scaldaferro, M. A. and Barboza, G. E. (2023). Cytogenetic characterization and chromosome evolution of the striking tropical Andean species of *Capsicum* (Solanaceae). *Plant Biosyst.* 157, 584–593. doi: 10.1080/11263504.2023.2166619

[3] Park, Y. K., Kim, B. D., Kim, B. S., Armstrong, K. C., and Kim, N. S. (1999). Karyotyping of the chromosomes and physical mapping of the 5S rRNA and 18S–26S rRNA gene families in five different species in *Capsicum*. *Genes Genet. Syst.* 74, 149–157. doi: 10.1266/ggs.74.149

[4] Park, Y. K., Park, K. C., Park, C. H., and Kim, N. S. (2000). Chromosomal localization and sequence variation of 5S rRNA gene in five *Capsicum* species. *Mol. Cells* 10, 18–24. doi: 10.1007/s10059-000-0018-4

[5] Scaldaferro, M. A., Seijo, J. G., Acosta, M. C., Barboza, G.E., Ducasse, D. A., and Moscone, E. A. (2006). Genomic characterization of the germplasm in peppers (*Capsicum*–Solanaceae) by fluorescent *in situ* hybridization. *Plant Sci.* 43, 291–297.

[6] Kwon, J. K., and Kim, B. D. (2009). Localization of 5S and 25S rRNA genes on somatic and meiotic chromosomes in *Capsicum* species of chili pepper. *Mol. Cells* 27, 205–209. doi: 10.1007/s10059-009-0025-z

[7] Scaldaferro, M. A., Romero da Cruz, M. V., Cecchini, N. M., and Moscone, E. A. (2016). FISH and AgNor-mapping of the 45S and 5S rRNA genes in wild and cultivated *Capsicum* species (Solanaceae). *Genome* 59, 95–113. doi: 10.1139/gen-2015-0099

[8] Aguilera, P. M., Debat, H. J., Scaldaferro, M. A., Martí, D.A., and Grabiele, M. (2016). FISH-mapping of the 5S rDNA locus in chili peppers (*Capsicum*-Solanaceae). *Genetica* 144, 653–662. doi: 10.1007/s10709-016-9940-1.

[9] Scaldaferro, M. A., and Moscone, E. A. (2019). "Cytology and DNA content variation of *Capsicum* genomes" in *Compendium of Plant Genomes*, eds N. Ramchiary and C. Kole (Cham, Switzerland: Springer Nature), pp. 57–84. doi: 10.1007/978-3-319-97217-6_4

[10] Belletti, P., Marzachi, C., and Lanteri, S. (1998). Flow cytometric measurement of nuclear DNA content in *Capsicum* (Solanaceae). *Plant Syst. Evol.* 209, 85–91. doi: 10.1007/BF00991526

[11] Moscone, E. A., Baranyi, M., Ebert, I., Greilhuber, J., Ehrendorfer, F., and Hunziker, A. T. (2003). Analysis of nuclear DNA content in *Capsicum* (Solanaceae) by flow cytometry and Feulgen densitometry. *Ann. Bot.* 92, 21–29. doi: 10.1093/aob/mcg105

[12] Jarret, R. L., Barboza, G. E., da Costa Batista, F. R., Berke, T., Chou, Y.-Y., Hulse-Kemp, A., Ochoa-Alejo, N., Tripodi, P., Veres, A., Carrizo García, C., Csillery, G., Huang, Y. K., Kiss, E., Kovacs, Z., Kondrak, M., Arce-Rodriguez, M. L., Scaldaferro, M. A., and Szoke, A. (2019). *Capsicum*: An abbreviated compendium. *J. Am. Soc. Hortic. Sci.* 144, 3–22. doi: 10.21273/JASHS04446-18
