# Supplementary material for: Patterns of base chromosome numbers, genome size and rDNA loci evolution in the genus Capsicum (Solanaceae)
Source: Front Plant Sci. 2026 Sep 28;17:1959260. doi: 10.3389/fpls.2026.1959260 (PMC13655049; doi:10.3389/fpls.2026.1959260)
Supplement: Supplementary file 3 [file DataSheet3.pdf]

Supplementary Data

Supplementary data 1.

Comparison of evolutionary models used for ancestral state reconstructions.

A. Genome size

Akaike Information Criteria weights

BM	OU	EB
0.4427966	0.1208352	0.4363683

> Best-fitting model = BM

B. Base chromosome number

Akaike Information Criteria weights

ER	Irr-1	Irr-2	ARD
0.3021526	0.3727309	0.2112432	0.1138732

> Best-fitting model = Irr-1

C. 35S rDNA loci number

Akaike Information Criteria weights

ER	MER	ARD	SYM
0.78856882	0.05789185	0.00812458	0.14541474

> Best-fitting model = ER

Supplementary data 2.

Comparison of evolutionary models used for ancestral state reconstructions of (A) base chromosome number (states 12 and 13) and (B) 35SrDNA loci numbers (states 1, 2 and ≥ 3 - represented by 3). Transition rates are specified by the arrows, which indicate the direction/s of change. The red stars indicate the best-fitting model.

A.

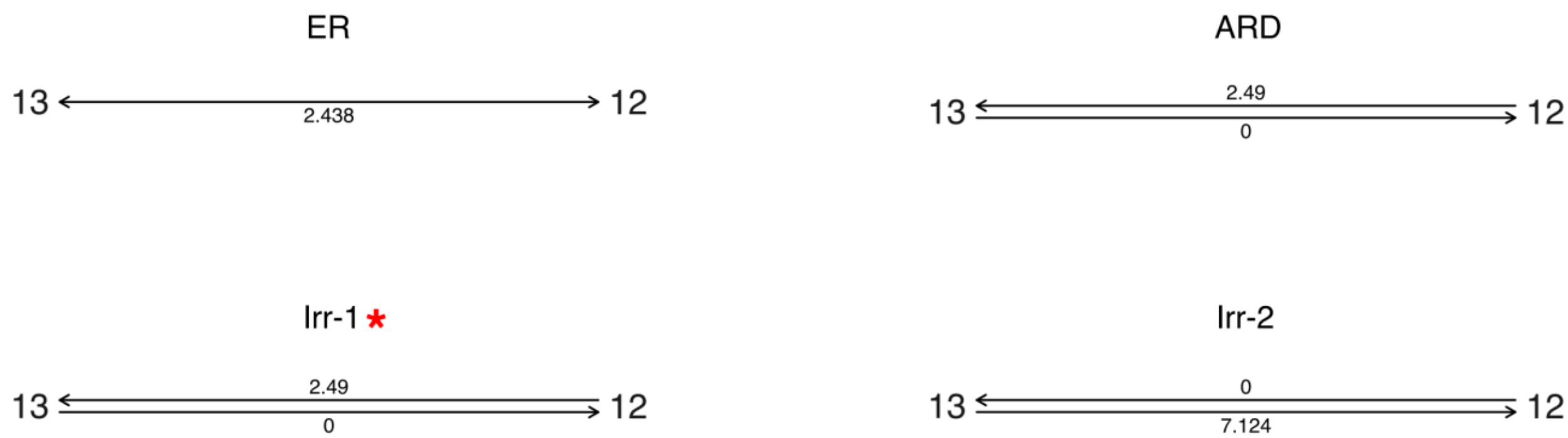

B.

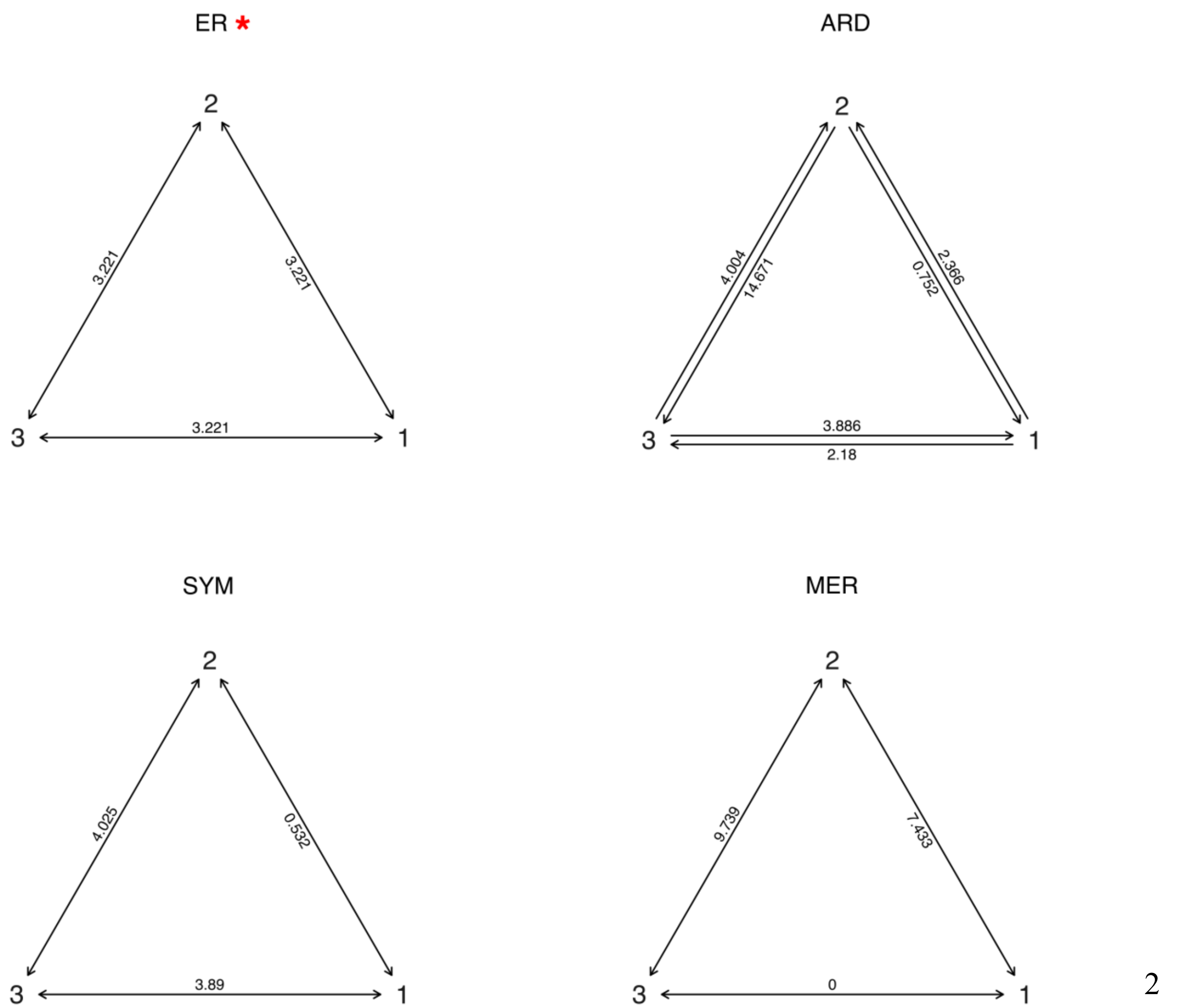

Supplementary data 3.

Phylogenetic signal recovered in ancestral state reconstructions of (A) genome size, (B) base chromosome number, and (C) 35SrDNA loci numbers.

A. Genome size
Phylogenetic signal K: 2.47196
P-value (based on 1000 randomizations): 0.001

Phylogenetic signal lambda: 0.999927
logL(lambda): -26.2511
LR(lambda=0): 48.6695
P-value (based on LR test): 3.02934e-12

B. Base chromosome number
Estimated D statistic: -0.8941745
Probability of E(D) resulting from no (random) phylogenetic structure: 0
Probability of E(D) resulting from Brownian phylogenetic structure: 0.906

Phylogenetic heritability H^2
Mode (most likely estimation): 0.999655914281978
95% highest posterior density - lower: 0.998296736501027
95% highest posterior density - upper: 0.999891013072792

C. 35S rDNA loci number
Phylogenetic heritability H^2
Mode (most likely estimation): 0.99740233674425
95% highest posterior density - lower: 0.900376016778744
95% highest posterior density - upper: 0.999534916551216

Supplementary data 4.

Results of base chromosome number reconstruction using ChromEvol.

Best-fitting model: Dys

Nodes with GAIN events with expectation above 0.5

Node 12 (Andean clade): 0.997171
Node 34 (Atlantic Forest clade): 0.994804
Total number of gain events: 2

Nodes with LOSS events with expectation above 0.5

Total number of loss events: 0

Nodes with duplication events with expectation above 0.5

Total number of duplication events: 0

Nodes with demi-duplication events with expectation above 0.5

Total number of demi-duplications events: 0

Nodes with transitions in base number events with expectation above 0.5

Total number of transitions in base number: 0

# EVENTS EXPECTATIONS PER NODE (main nodes/clades)

Clades based on $x = 13$ marked in red (Andean clade and Atlantic Forest clade). Remaining clades are based on $x = 12$.

NODE	GAIN	LOSS	$x = 12$	$x = 13$
Capsicum	5.72306e-06	0	**0.999999**	3.84495e-11
**Andean clade**	**0.997171**	0	0.00283027	**0.99717**
Flexuosum + Caatingae clades	1.79446e-11	0	**1**	0
Caatinga clade	4.74424e-12	0	**1**	0
**Atlantic Forest clade**	**0.994804**	0	0.00519649	**0.994804**
Bolivian clade	2.53274e-10	0	**1**	0
Pubescens clade	2.27839e-12	0	**1**	0
Tovarii + Baccatum + Annuum clades	2.36435e-12	0	**1**	0
Baccatum clade	2.09342e-15	0	**1**	0
Annuum clade	2.48428e-15	0	**1**	0
